# Prevalence of Electronic Cigarette Use and Its Determinants among 13-to-15-Year-Old Students in Greece: Results from the 2013 Global Youth Tobacco Survey (GYTS)

**DOI:** 10.3390/ijerph17051671

**Published:** 2020-03-04

**Authors:** Soteris Soteriades, Anastasia Barbouni, George Rachiotis, Panoraia Grevenitou, Varvara Mouchtouri, Ourania Pinaka, Katerina Dadouli, Christos Hadjichristodoulou

**Affiliations:** 1 Laboratory of Hygiene and Epidemiology, Faculty of Medicine, University of Thessaly, Katsigra Building, 22 Papakyriazi St., 41222 Larissa, Greecexhatzi@med.uth.gr (C.H.); 2 Department of Public Health, University of West Attica, 196 Alexandras Ave., 11521 Athens, Greece

**Keywords:** adolescents, tobacco smoking, vaping, e-cigarette, nicotine, gateway, susceptibility, prevalence, GYTS, Greece

## Abstract

Electronic cigarette use has increased over the past decade. Its potential role in smoking cessation, in addiction and as a ‘gateway’ to tobacco smoking is subject to intense research. This cross-sectional study, carried out in 2013, aims to present the habits of students aged 13–15 in Greece with regard to e-cigarettes and investigate potential risk factors and the relationship between e-cigarettes, tobacco smoking and other nicotine products. It is the first such study to be carried out in Greece. The survey was based on the standardized methodology of the Global Youth Tobacco Survey. All potential associations were investigated using multiple logistic regression. In total 2.8% of the 4096 participating students were current e-cigarette users and 12.3% of them were ever users. E-cigarette use was associated with male gender, being older, current use of combustible tobacco products and e-cigarette use in the family. Ever e-cigarette use, being older, female gender and higher pocket money were associated with an increased susceptibility to tobacco smoking. E-cigarette prevalence in Greece in 2013 was similar to that of other, developed countries. The smoking and vaping habits of adolescents must be monitored in order to assess trends over time and whether any policy alterations are necessary.

## 1. Introduction

E-cigarettes are battery-operated nicotine delivery devices consisting of a battery-powered system, a liquid container, an atomizing device and an inhaler that simulates the filter of conventional cigarettes [1]. The atomizing device heats the liquid and transforms it into an inhalable vapor [2] that typically contains nicotine. E-cigarette liquid contains a variety of toxic components, albeit at far lower quantities compared to tobacco smoke and often comparable to the trace amounts present in pharmaceutical nicotine inhalators [3,4,5]. Electronic cigarettes do not contain tobacco and do not produce smoke, since no combustion occurs; however, they are recognized as tobacco products by the regulatory authorities of many countries [6]. They come in a variety of flavors such as coffee, vanilla or other sweets, mint and fruits, increasing its appeal to youth in particular. It has been suggested that e-cigarettes may be used for smoking cessation and they are promoted as a safer alternative to tobacco smoking, but uncertainty surrounds their potential long-term health effects. A report [7] commissioned by Public Health England found that e-cigarettes may pose less than 0.5% of the cancer risk posed by tobacco smoking, but that risks of cardiovascular and lung disease have not been quantified; therefore, further investigation is required regarding the long-term health effects of e-cigarettes. Overall, the report estimates that e-cigarette vaping causes 95% less harm than tobacco smoking [7].

There is evidence that nicotine-containing e-cigarettes increase the risk of nicotine dependence. In particular, it has been reported that exposure to nicotine during adolescence can cause addiction and harm the developing adolescent brain [8]. Because of this, many claim that e-cigarettes are a ‘gateway’ to tobacco smoking. Studies have found that e-cigarette use among students is associated with susceptibility to conventional cigarettes, although the direction of causality is not clear [9]. Based on this ‘gateway hypothesis,’ it is speculated that drug users go through a sequence of drug initiation, starting from milder and moving to stronger drugs. Use of the milder drug is considered a necessary condition in order to move on to the next one. In this particular case, the claim is that e-cigarette use is causally linked to the initiation of tobacco smoking [10,11,12]. Others authors [13] have pointed out that many of these studies are cross-sectional, thus, it cannot be determined whether susceptibility to smoking precedes the use of e-cigarettes or vice versa. There is also evidence to support a ‘common liability’ hypothesis: all addictions are influenced by a unidimensional liability trait, which is multifactorial but nevertheless a non-specific predictor of any addiction development. In this case, e-cigarette use and tobacco smoking can be viewed as two independent results of an underlying common liability to addiction [14,15,16]. The ‘catalyst model’ [17] provides a comprehensive account of numerous factors that may influence the relationship between e-cigarette use and tobacco smoking. Susceptibility to smoking may be what leads adolescents to experiment with e-cigarettes or vice versa. A study using data from the WHO World Mental Health Survey [18] found that future development of dependence upon a drug is not affected by the order of previous drug initiation but by the extent of prior use of any drug and the age of onset at which that use began.

On the other hand, certain prospective cohort studies [19,20,21,22] of adolescents have measured the risk of initiating tobacco smoking between e-cigarette users and non-users. The calculated risk is adjusted in each study for various presumed confounding factors. However, liability to addiction, as a risk factor itself, is not investigated in any of these studies. Due to the study design, individuals were excluded from the sample if they were tobacco smokers at the start of the study. This means that any positive effect of e-cigarette use on tobacco smoking prevalence cannot be observed, such as smokers who quit or reduced their use of combustible tobacco products. Furthermore, the outcome under investigation in these studies was the ever use of tobacco cigarettes (“at least one puff”), while the frequency and extent of tobacco smoking was not included in the analysis. Criticism of e-cigarettes also concentrates on the fact that they damage cells, they increase the risk of chronic diseases, they increase the risk of smoking and they generally contain harmful chemicals [23]. The studies on which such criticism relies often do not compare the harm of e-cigarettes to tobacco smoke [24] or, as has been mentioned previously, rely on cross-sectional analyses [25], which cannot support causal inferences. However, talking in general of “harm” and “risk” is not constructive. Multiple aforementioned studies have quantified these risks and estimated that tobacco smoke is substantially more harmful than electronic cigarettes. Another salient point on the issue, noted by the World Health Organization (WHO) [26], is that it is inaccurate to speak of the risk of vaping as the risk of one single product. The risk can depend on “the brand and batch […], the preferred flavor, the heating of the e-cigarette, the vaporizer, how dirty or worn the e-cigarette is, the method of vaping, and factors still unknown”.

In light of the above, the first important step is to establish the prevalence of use of combustible tobacco products and e-cigarettes, the variation of prevalence over time and the associated risk factors. Unfortunately, the literature on the prevalence of e-cigarette use by Greek adolescents is lacking. One study [27], using data from WHO’s 2014 Health Behaviour in School-aged Children study, performed a preliminary cross-sectional analysis of nationally representative data collected only from 15-year-old students in Greece, in 2014. It found that 16.6% of 15-year-olds had ever used an e-cigarette and 0.8% of them were currently using it. The present study aimed to enrich our knowledge on this issue. It was carried out earlier, in 2013, by means of a Global Youth Tobacco Survey (GYTS) questionnaire, which is a school-based survey that collects data on students 13–15 years using a standardized methodology for constructing the sampling frame, selecting schools and classes, preparing questionnaires, following consistent field procedures and using consistent data management procedures for data processing and analysis [28]. Other aims of the current study were to estimate the prevalence of susceptibility to tobacco smoking among e-cigarette users and non-users and to determine the risk factors that may influence e-cigarette use.

## 2. Materials and Methods

### 2.1. Data Collection

A cross-sectional, voluntary, school-based, self-administered, pencil-and-paper survey, based on the GYTS questionnaire, was conducted in public and private schools, in Athens and in the rest of the country. The target population was students aged 13–15 years, both boys and girls. Any children that were younger or older were excluded from the analysis. The questionnaire contained several questions on demographic information for the students and their families, questions on tobacco smoking, on use of other tobacco products, on use of e-cigarettes and on the use of such products by other family members. Furthermore, it contained questions pertaining to addiction, exposure to tobacco advertising and awareness of the health effects of tobacco and other nicotine-containing products.

### 2.2. Variables

All variables were produced based on the questionnaire that is included in the Supplementary Material. With regard to the predictors, the aim was to convert information from the questionnaire into binary variables. The exceptions were age, which had three categories (13, 14 and 15 years old), and pocket money per week, which had four. Overall, four types of nicotine-containing products were asked about: tobacco cigarettes, other combustible tobacco products (pipe, cigars, cigarillos, hookahs/shisha), non-combustible tobacco products (chewing tobacco or tobacco candy) and e-cigarettes. These products were also categorized into combustible tobacco products, which included the first two product types, and non-combustible nicotine-containing products, which contained the last two types. Experimentation with (i.e., ever use of) e-cigarettes was defined as answering “yes” to the question: “Have you ever tried or experimented with the electronic cigarette?” The same question was asked for ever use of other nicotine product types. Current e-cigarette use was defined as answering “Yes” to the question “During the past 30 days, have you used the electronic cigarette?” Similar questions were asked to establish current use of other nicotine product types. Smoking in the family was defined as answering anything other than “No one” to the question “Who smokes in the house?” Possible answers included: father, mother, siblings, both parents, more than two people. Other predictor variables that were investigated included gender, age, pocket money per week, paternal and maternal educational levels, tobacco smoking in the family and e-cigarette use in the family.

There were two outcome variables. Current e-cigarette use was defined as answering “yes” to the question: “During the past 30 days, have you used the electronic cigarette?” Susceptibility to tobacco smoking was defined as answering anything other than “definitely not” to the question “If one of your best friends offered you a cigarette, would you smoke it?” or anything other than “definitely not” to the question “At any time during the next 12 months, do you think you will use any form of tobacco?” For this particular outcome, all students who had reported ever use of tobacco cigarette, other combustible tobacco products or non-combustible tobacco products were excluded from the analysis. It should be noted that students who reported ever use of e-cigarettes (but no use of other nicotine-containing products) were not excluded.

### 2.3. Sampling

Multi-stage clustered sampling was carried out, with each school having a likelihood of being selected proportional to the number of enrolled students. There were two geographical strata: the Attica region (which contains Athens) and the rest of Greece. The Primary Sampling Units (PSUs) were 30 high schools from each stratum. The selection of classes within the schools was done by simple random sampling. The final sampling units consisted of all students in the selected classes who were attending school on the day of the survey.

### 2.4. Ethics

Parents were notified by a letter and students gave verbal consent to participate in the study. The protocol of the study was approved by the Ministry of Education and Religious Affairs and by the Institutional Review Board of the National School of Public Health (NSPH) in Athens, Greece (ID: 41996/Γ2/6-4-2011 and 41996/Γ2/31-5-2012).

### 2.5. Statistical Analysis

Prior to any analysis, a weighting factor was applied to reflect the probability of sampling of each student. The weight was calculated with this formula: W_i_ = W1 × W2 × f1 × f2 × f3 × f4, where

- W1: the inverse of the probability of selecting the school
- W2: the inverse of the probability of selecting the classroom within the school
- f1: a school-level non response adjustment factor calculated by school size category (small, medium, large)
- f2: a class-level non response adjustment factor calculated for each school
- f3: a student-level non-response adjustment factor calculated by class
- f4: a post-stratification adjustment factor calculated by grade.

Individual weights were then scaled down, so that the size of the sample would remain the same for the purposes of the statistical analysis. Therefore, the final weight for each participant was calculated using the following formula:

W_final_ = $\frac{W_{i}\times n}{\sum_{i=1}^{n}W_{i}}$, where W_i_ is the initial weight calculated for each participant, *n* is the total number of participants (4096) and $\sum_{i=1}^{n}W_{i}$ is the sum of all initial weights.

After applying the weights, a frequency analysis was performed. Furthermore, the prevalence of each type of nicotine-containing product was determined. Then, two multivariable models were developed. Current e-cigarette use was the outcome of the first model and susceptibility to smoking was the outcome of the second model. For each model, a bivariate analysis was first carried out using Pearson’s chi-square test. Or, if more than 25% of the expected counts were less than 5, Fisher’s exact test was used instead. If the correlation between a predictor and the outcome had a *p*-value of less than 0.1, the predictor was included in the multivariable analysis. The multivariable analysis was carried out using multiple binary logistic regression, including an intercept value. Entries with missing data were not taken into account. IBM SPSS Statistics for Windows, Version 25.0 (IBM Corp., Armonk, NY, USA) was used to carry out all statistical tests. All statistical tests were performed at a two-tailed significance level of 0.05. Point estimates are accompanied by a 95% confidence interval (95% CI).

## 3. Results

A total of 5127 students were selected to participate, of which 4618 responded. Taking into account the school response rate, overall response rate was 87.7%. Of these, 4096 respondents were 13–15 years old. In terms of gender distribution, 51.5% were boys and 48.5% were girls. The prevalence of experimentation with e-cigarettes was estimated at 12.3% (95% CI: 11.2–13.3%). Current e-cigarette use was at 2.8% (95% CI: 2.3–3.3%). The prevalence of current use of tobacco cigarettes was 10.1% (95% CI: 9.1–11.0%) and ever use was 30.4% (95% CI: 11.2–13.3%). The frequency of each characteristic among all respondents is shown in Table 1, with the corresponding 95% confidence interval.

In Table 2, we can compare the prevalence of nicotine-containing products by type (the 95% confidence interval for each proportion is found in brackets). It includes data for both ever use and current use. The category “any combustible product” included tobacco cigarettes and other combustible tobacco products (rolled cigarettes, pipes, cigars, cigarillos, hookahs), while the category “any nicotine-containing product” contained all product types. In all cases, current use increased with age. Tobacco cigarettes were the most common product type. Since the sum of individual product type prevalence was bigger than the prevalence of “any nicotine-containing product”, it is clear that many students who used nicotine-containing products used more than one type of product. In fact, based on the data of the survey, 5.7% of students were current users of more than one type of product (95% CI: 5.0–6.4%). Among current users of at least one type of product, nearly half (42.4%, 95% CI: 38.2–46.5%) currently used more than one type of product.

In Table 3, the results of the bivariate analysis are presented. It was found that the prevalence of e-cigarette use was 3.9% among boys and 1.7% among girls. There was a statistically significant increasing trend of current e-cigarette use with higher age, since the prevalence was 1.1%, 3.2% and 4.7% for age 13, 14 and 15, respectively. Other factors that seemed to be significantly positively associated with e-cigarette use include: current use of any combustible tobacco product, e-cigarette use by other family members and lower paternal education level. Maternal education level, pocket money per week and tobacco smoking in the family were not associated with e-cigarette use. No odds ratio (OR) is shown for age and pocket money per week, since Pearson chi-square tests only produce an OR for binary variables. It was decided that, although tobacco smoking in the family was close to statistical significance, it would not be included in the multiple regression model, because the “No” group contained only 14 respondents, while the “Yes” group contained 4041 respondents. Therefore, any analysis containing this variable would be unreliable. For age and pocket money per week, the OR and 95% CI were not applicable (N/A).

The multivariable analysis (Table 4) shows that male gender, increasing age, current use of any combustible tobacco product and e-cigarette use in the family were independently associated with current e-cigarette use. Boys have 2.56 times the odds of being electronic-cigarette users compared to girls (95% CI: 1.56–4.20). Both 14 and 15-year-olds had significantly higher adjusted odds of using e-cigarettes than 13-year-olds, with an adjusted odds ratio (aOR) of 2.01 (95% CI: 1.03–3.92) and 2.87 (95% CI: 1.48–5.56), respectively. Current users of combustible tobacco products had 7.85 times the odds of being e-cigarette users compared to non-users (95% CI: 5.00–12.3). E-cigarette use in the family was also a strong predictor (aOR: 5.72, 95% CI: 3.65–8.97). Finally, paternal education level was not found to be significantly associated with e-cigarette use. All covariates (predictors) included in the model can be seen in Table 4.

In the last part of the analysis, data from never-smokers were analyzed in order to investigate susceptibility to tobacco smoking and its relationship to e-cigarette use.

Table 5 shows the results of the bivariate analysis of risk factors that are potentially associated with susceptibility to smoking. These were investigated by restricting the analysis to students who had never used any tobacco products (except e-cigarettes). The proportion of boys that were susceptible to smoking was found to be 11.3%. The corresponding proportion for girls was 14.4%. Susceptibility to tobacco smoking also increased with age since the prevalence was 9.6%, 15.4% and 15.5% among ages 13, 14 and 15, respectively. Furthermore, a higher amount of pocket money per week and ever e-cigarette use was also significantly associated with susceptibility to smoking. On the other hand, higher paternal and maternal education levels, as well as tobacco smoking in the family and e-cigarette use in the family, were far from reaching statistical significance, thus, they were not included in the multivariable model.

The results of the multivariable analysis are shown in Table 6. Girls had a significantly higher susceptibility to smoking compared to boys (aOR for boys to girls: 0.72, 95% CI: 0.57–0.92). Compared to 13-year-old students, both 14- and 15-year-olds had higher odds of being susceptible to smoking, with an aOR of 1.61 (95% CI: 1.22–2.12) and 1.63 (95% CI: 1.19–2.22), respectively. Furthermore, having 10–19 € or more than 20 € to spend per week were also associated with higher susceptibility to smoking, compared with having no money to spend. Respectively, aOR was 2.41 (95% CI: 1.28–4.53) and 2.24 (95% CI: 1.10–4.58). Finally, ever e-cigarette use was also found to be a statistically significant predictor of susceptibility to tobacco smoking, with an aOR of 3.06, (95% CI: 1.73–5.41). All covariates (predictors) included in the model can be seen in Table 6.

## 4. Discussion

### 4.1. Main Findings and Comparison with Other Studies

To our knowledge, this is the first nationwide cross-sectional study performed in Greece that investigated the prevalence of electronic cigarette use among students aged 13–15 years old. The main findings are that, in 2013, 2.8% of all students in Greek middle schools were current e-cigarette users and 12.3% of students had experimented with e-cigarettes at least once. E-cigarette users in this study were far more likely to be male, older (15 vs. 13 years old), users of combustible tobacco products or members of families where other members also used e-cigarettes—the latter two variables were the strongest predictors. Parental education level and amount of pocket money received per week were not associated with e-cigarette usage status. However, for students that had used multiple nicotine product types, the sequence of initiation was not determined. It was also not determined whether students started using e-cigarettes before or after their other family members.

Many studies estimated that prevalence of e-cigarette usage among adolescents in 2013 reached 1.1–2.9% in the US (estimates differ due to methodological variation) [29,30]. The National Youth Tobacco Survey (NYTS), which is performed annually in the US, applied the same methodology as GYTS. It was found that there was a non-linear increase of e-cigarette users among middle-school students from 0.6% in 2011 to 4.9% in 2018 [31]. Increases in point-prevalence of e-cigarette use have been observed in other countries as well, such as Italy (from 8.4% in 2014 to 17.5% in 2018) and Poland (from 5.5% in 2010–2011 to 29.9% in 2013–2014) [32,33]. It is possible that similar increases may have also occurred in Greece; therefore, it is important to repeat GYTS, in order to assess changes and trends in prevalence after 2013.

The current study’s findings are consistent with other cross-sectional and cohort studies [34,35,36], which have found that male gender and prior addictive substance use (including tobacco smoking) is positively associated with e-cigarette use. Other cross-sectional studies [37] have also found that smoking in the family is positively associated with student e-cigarette use, whereas the current study found that e-cigarette use in the family is a predictor of student e-cigarette use. Overall, the temporal sequence between the presumed predictors and outcomes is unclear; nevertheless, the evidence supports the idea that family members influence each other’s habits with regard to the use of nicotine-containing products. Furthermore, a staggering 99.5% of participants in the study reported that at least one other family member smokes (parent or sibling). Because the number of children with no smoking in the family was only 14, the results for this variable are not very reliable and it is not possible to exclude the possibility that family smoking is indeed a risk factor associated with e-cigarette use. Of note is the high ratio (4.4) of ever use to current use of e-cigarettes, a ratio that does not exceed 3.0 for any other product. This suggests that many students experiment with e-cigarettes, but few make it into a habit. One of many possible explanations is that those who experimented with e-cigarettes did so by using a friend’s device instead of buying their own.

Furthermore, the study found that, among never smokers, susceptibility to tobacco smoking was positively associated with higher age, female gender, higher amounts of pocket money and ever e-cigarette use. These results have many possible interpretations. With regard to the influence of gender and age on susceptibility to smoking, many studies [38,39,40,41] have taken them into account, but the results have been mixed, with some finding a statistically significant association and others that did not. These studies’ populations have different age distributions and come from different countries with very different cultural backgrounds, thus, it is difficult to draw conclusions about gender and age in general. The findings with regard to pocket money are also consistent with other studies [42]. With regard to the influence of e-cigarettes, since this study is cross-sectional, it is not possible to determine whether susceptibility to tobacco smoking arose after the use of e-cigarettes or pre-existed.

Many studies, mentioned in the introduction, have investigated whether e-cigarette use increases the risk of students’ taking up tobacco smoking. In addition to those studies, a prospective observational cohort study [43] in the United Kingdom was carried out among students aged 13–14 in 2014 who had never used either conventional or electronic cigarettes. Two years later, 25.2% of them had ever used e-cigarette and 15.3% of them had ever used conventional cigarettes. Each of the above groups included dual users of electronic and conventional cigarettes. Those who smoked tobacco regularly (1.2%) were far more likely to be ‘dual users–tobacco used first’ rather than exclusive users of tobacco or ‘dual users–e-cigarettes used first’. If the ‘gateway’ hypothesis were true, one would expect a disproportionately higher number of dual users to have started e-cigarettes first and then progressed to tobacco smoking, not vice versa. Furthermore, a cross-sectional study carried out in the US based on 2011–2013 data from the National Youth Tobacco Survey (NYTS) for grades 6–12 found that, between 2011 and 2013, intention to smoke tobacco cigarettes decreased significantly despite a three-fold increase in ever use of e-cigarettes among non-smokers [9]. Another study, based on 2011–2018 NYTS data for middle-school students, found that total use of nicotine-containing products had not changed significantly over this 7-year period (7.5% on 2011 to 7.2% in 2018), but e-cigarette use (up from 0.6% to 4.9%) had significantly replaced the use of combustible tobacco products (down from 6.4% to 3.3%) during this period [31]. All in all, it seems that e-cigarette use may contribute to a *net* reduction in the use of combustible tobacco products among adolescent students.

The current study’s results neither affect this balance of evidence nor contradict the existing evidence. The results from the above studies strongly indicate that the point-prevalence is not sufficient to draw conclusions about the effect of adolescents’ e-cigarette use on tobacco smoking and that the changes observed over time must be taken into account. Since any use of nicotine-containing products in children and adolescents is unsafe, it is important to minimize the harm caused by the use of these products. In this sense, it seems counterproductive to selectively restrict adolescents’ access to e-cigarettes compared to combustible tobacco products, since this may result in more adolescents using combustible tobacco products, which are more harmful. The current study’s results suggest that, among Greek adolescents, almost half of current nicotine product users use more than one product. This is another indication that selectively targeting e-cigarettes would not be effective. Alternatively, in order to mitigate the health effects of nicotine product use by adolescents, it is important to adopt an integrated approach which quantifies the total harm caused by nicotine products and identifies the ideal ways to minimize it.

### 4.2. Limitations

There are certain limitations concerning the current study. Since it was cross-sectional, causal inferences cannot be made with regard to the associations found between certain risk factors and the outcomes. The response rate was sufficiently high, but we cannot exclude the possibility of selection bias if refusal to participate was related to e-cigarette use or smoking status. Therefore, it is possible that prevalence of nicotine product use was under-reported. Secondly, since we rely on self-reported data, there may be recall bias or social desirability bias, with students being reluctant to admit the use of nicotine products. Furthermore, not all children in Greece aged 13–15 are enrolled in school. E-cigarette use may have a different prevalence among children who do not attend school. For the above reasons, it is speculated that the true prevalence of e-cigarette vaping among 13–15-year-old adolescents in Greece is higher than estimated.

Comparison of this study with others can be made difficult by differences in the wording of questions that the students were asked. This can affect how they are answered. For example, some studies distinguish between daily use and non-daily use of e-cigarettes, while others, such as this one, distinguish between ever use and current use [44]. Current use has been defined by different studies as past-30-day prevalence, as past-5-day prevalence, as more than one cigarette per week or as “fairly regular” use. Efforts should be made to achieve greater homogeneity between studies with regard to the definitions and the wording of each question.

### 4.3. Further Research Directions

Future research could take into account e-cigarettes that do not contain nicotine and e-cigarettes that contain other addictive substances, since e-cigarettes can also be used as illicit drug delivery systems [45]. Future studies could also attempt to determine the frequency of use among current users of e-cigarettes and study potential misconceptions about the safety of e-cigarettes among the general public and adolescents in particular [46,47]. Finally, point-prevalence studies such as the current one should be repeated, in order to observe long-term trends.

## 5. Conclusions

The study found that 2.8% of Greek students aged 13–15 were current users of e-cigarettes in 2013 and 12.3% were ever users. Associated risk factors included being male, being older, current use of combustible tobacco products and e-cigarette use in the family. These findings provide extensive and valuable background information on the prevalence of use of e-cigarettes and its predictors among adolescents in Greece. Susceptibility to tobacco smoking was associated with higher age, female gender, higher amounts of pocket money and ever e-cigarette use. However, since this study is cross-sectional, causal inferences could not be made. The prevalence of e-cigarette use among Greek adolescents is likely to have increased since 2013, since such increases have been observed in other countries during the last decade. For policy recommendations to be made, a comprehensive population-level risk-benefit analysis of e-cigarettes would require up-to-date information on prevalence, relationship with tobacco smoking and determination of associated risk factors.

## Figures and Tables

**Table 1:** Descriptive table of frequencies including all participants.

Frequencies				
		%	95% CI	
Current use of e-cigarettes	Yes	2.8%	2.3%	3.3%
	No	97.2%	96.7%	97.7%
Ever use of e-cigarettes	Yes	12.3%	11.2%	13.3%
	No	87.7%	86.7%	88.8%
Gender	Boy	51.5%	50.0%	53.0%
	Girl	48.5%	47.0%	50.0%
Age	15 years	27.4%	26.0%	28.8%
	14 years	35.0%	33.5%	36.4%
	13 years	37.7%	36.2%	39.1%
Pocket money per week	≥20 €	10.7%	9.7%	11.6%
	10–19 €	28.5%	27.1%	29.8%
	1–9 €	55.0%	53.4%	56.5%
	No money to spend	5.9%	5.2%	6.6%
Education level of father	At least high school	72.2%	70.8%	73.6%
	Until junior high school	27.8%	26.4%	29.2%
Education level of mother	At least high school	78.1%	76.8%	79.4%
	Until junior high school	21.9%	20.6%	23.2%
In your family, does anyone smoke?	Yes	99.5%	99.3%	99.7%
	No	0.5%	0.3%	0.7%
Susceptibility to tobacco cigarettes	Yes	12.9%	11.6%	14.2%
	No	87.1%	85.8%	88.4%
Current use of tobacco cigarettes	Yes	10.1%	9.1%	11.0%
	No	89.9%	89.0%	90.9%
Ever use of tobacco cigarettes	Yes	30.4%	29.0%	31.9%
	No	69.6%	68.1%	71.0%
In your family does anyone use the electronic cigarette?	Yes	12.3%	11.3%	13.3%
	No	87.7%	86.7%	88.7%

**Table 2:** Prevalence of nicotine product use by type in 2013.

Product Type	Any Nicotine-Containing Product	Any Combustible Product	Tobacco Cigarettes	Other Combustible Tobacco Product	Non-Combustible Tobacco Product	E-Cigarettes
Prevalence of ever use	38.0% (36.5–39.5%)	34.9% (33.4–36.4%)	30.4% (29.0–31.9%)	16.7% (15.6–17.9%)	3.4% (2.8–3.9%)	12.3% (11.2–13.3%)
Prevalence of current use	15.2% (14.1–16.4%)	13.3% (12.2–14.3%)	10.1% (9.1–11.0%)	7.6% (6.8–8.4%)	1.5% (1.1–1.9%)	2.8% (2.3–3.3%)
Current use (13 years old)	7.3% (5.9–8.6%)	5.7% (4.5–6.9%)	4.7% (3.6–5.8%)	2.8% (1.9–3.6%)	1.0% (0.5–1.5%)	1.1% (0.6–1.6%)
Current use (14 years old)	16.3% (14.4–18.3%)	14.2% (12.3–16.0%)	10.5% (8.9–12.2%)	8.6% (7.1–10.1%)	1.6% (1.0–2.3%)	3.2% (2.3–4.2%)
Current use (15 years old)	24.4% (21.9–27.0%)	22.4% (19.9–24.9%)	16.9% (14.7–19.1%)	13.0% (11.0–15.0%)	2.1% (1.2–2.9%)	4.7% (3.4–5.9%)

**Table 3:** Bivariate analysis of current e-cigarette use (all participants).

Predictor		Outcome: Current E-Cigarette Use			
		Proportion	OR	95% CI	*p*-Value
	Total	2.8%	–	–	–
Gender	Boy	3.9%	2.40	1.60–3.62	<0.001
	Girl	1.7%	ref.		
Age	15 years	4.7%	N/A	N/A	<0.001
	14 years	3.2%			
	13 years	1.1%			
Pocket money per week	≥20 €	3.9%	N/A	N/A	0.159
	10–19 €	3.3%			
	1–9 €	2.3%			
	Usually I have no money to spend	2.1%			
Educational level of father	At least high school	2.5%	0.64	0.43–0.96	0.029
	Until junior high school	3.8%	ref.		
Educational level of mother	At least high school	2.7%	0.71	0.47–1.09	0.112
	Until junior high school	3.7%	ref.		
Tobacco smoking in the family	Yes	2.8%	0.17	0.04–0.77	0.057
	No	14.3%	ref.		
Any combustible tobacco product (current use)	Yes	12.5%	11.6	7.73–17.3	<0.001
	No	1.2%	ref.		
E-cigarette use in the family	Yes	10.2%	6.45	4.38–9.45	<0.001
	No	1.7%	ref.		

**Table 4:** Multivariable analysis of e-cigarette use (all participants).

Predictors		Outcome: Current E-Cigarette Use		
		aOR	95% CI	*p*-Value
Gender	Boy	2.56	1.56–4.20	<0.001 *
	Girl	ref.		
Age	15 years	2.87	1.48–5.56	0.002 *
	14 years	2.01	1.03–3.92	0.041 *
	13 years	ref.		
Paternal education level	At least high school	0.792	0.499–1.26	0.323
	Until junior high school	ref.		
Any combustible tobacco product (current use)	Yes	7.85	5.00–12.3	<0.001 *
	No	ref.		
E-cigarette use in the family	Yes	5.72	3.65–8.97	<0.001 *
	No	ref.		
Intercept value: 3.56				<0.001 *

Statistically significant *p*-values are noted with an asterisk (*).

**Table 5:** Bivariate analysis of susceptibility to tobacco smoking among never smokers.

Predictors		Susceptibility to Tobacco Smoking			
		Proportion	OR	95% CI	*p*-Value
	Total	12.9%			
Gender	Boy	11.3%	0.75	0.59–0.95	0.018
	Girl	14.4%	ref.		
Age	15 years	15.5%	N/A	N/A	<0.001
	14 years	15.4%			
	13 years	9.6%			
Pocket money per week	≥20 €	16.8%	N/A	N/A	<0.001
	10–19 €	18.0%			
	1–9 €	10.6%			
	Usually I have no money to spend	7.7%	ref.		
Education level of father	At least high school	13.2%	1.14	0.85–1.53	0.390
	Until junior high school	11.8%	ref.		
Education level of mother	At least high school	13.5%	1.20	0.87–1.65	0.258
	Until junior high school	11.5%	ref.		
Tobacco smoking in the family	Yes	12.9%	0.59	0.07–5.30	0.498
	No	20.0%	ref.		
Ever e-cigarette use	Yes	31.1%	3.19	1.83–5.56	<0.001
	No	12.4%	ref.		
E-cigarette use in the family	Yes	14.7%	1.19	0.82–1.73	0.369
	No	12.7%	ref.		

**Table 6:** Multivariable analysis of susceptibility to tobacco smoking among never smokers.

Predictors		Outcome: Susceptibility to Tobacco Smoking		
		aOR	95% CI	*p*-Value
Age	15 years old	1.63	1.19–2.22	0.002 *
	14 years old	1.61	1.22–2.12	0.001 *
	13 years old	ref.		
Gender	Boy	0.72	0.57–0.92	0.008 *
	Girl	ref.		
Pocket money	≥20 €	2.24	1.10–4.58	0.026 *
	10–19 €	2.41	1.28–4.53	0.006 *
	1–9 €	1.38	0.74–2.56	0.311
	Usually I have no money to spend	ref.		
Ever e-cigarette use	Yes	3.06	1.73–5.41	<0.001 *
	No	ref.		
Intercept value: 6.026				<0.001 *

Statistically significant *p*-values are noted with an asterisk (*).

## Supplementary Materials

The following are available online at https://www.mdpi.com/1660-4601/17/5/1671/s1, File S1: GYTSEURO2013 Greece All Schools (National) Web Codebook 20151009_508tag, File S2: GYTS 2013 Greece (National) WEB, File S3: Output of statistical analysis.
