# Supplementary material for: Prevalence of Electronic Cigarette Use and Its Determinants among 13-to-15-Year-Old Students in Greece: Results from the 2013 Global Youth Tobacco Survey (GYTS)
Source: Int J Environ Res Public Health. 2020 Mar 4;17(5):1671. doi: 10.3390/ijerph17051671 (PMC7084902; doi:10.3390/ijerph17051671)
Supplement: Supplementary file 1 [file ijerph-17-01671-s001.zip › GYTSEURO2013 Greece All Schools (National) Web Codebook 20151009_508tag.pdf]

Original Question	Analysis Variable	Question Code and Label	Unweighted Frequency	Weighted Percent
Q1	CR1	How old are you?		
		. Missing	2	.
		1 11 years old or younger	3	0.1%
		2 12 years old	369	8.0%
		3 13 years old	1,528	33.4%
		4 14 years old	1,407	31.0%
		5 15 years old	1,161	24.3%
		6 16 years old	108	2.4%
		7 17 years old or older	40	0.9%
Q2	CR2	What is your sex?		
		. Missing	4	.
		1 Male	2,348	52.0%
		2 Female	2,266	48.0%
Q3	GRR3	In what grade/form are you?		
		. Missing	7	.
		1 First grade	1,593	34.9%
		2 Second grade	1,499	33.4%
		3 Third grade	1,519	31.7%
Q4	GRR4	How much money do you normally spend on your personal needs?		
		. Missing	21	.
		1 Usually I have no money to spend	299	6.0%
		2 Less than 5 €	1,197	26.0%
		3 5 to 9 €	1,345	29.2%
		4 10 to 14 €	818	17.8%
		5 15 to 19 €	445	9.9%
		6 20 to 24 €	231	5.3%
		7 25 to 29 €	107	2.4%
		8 More than 30 €	155	3.4%
Q5	OR1	Do your parents work?		
		. Missing	9	.
		1 Father (stepfather or mother's partner) only	1,291	27.4%
		2 Mother (stepmother or father's partner) only	387	8.3%
		3 Both	2,694	59.4%
		4 Neither	201	4.1%
		5 Don't know	36	0.8%
Q6	GRR6	What level of education did your father (stepfather or mother's partner) complete?		
		. Missing	17	.
		1 He's never gone to school	18	0.4%
		2 He has graduated from elementary school	401	9.6%
		3 He has graduated from junior high school	714	16.0%
		4 He has graduated from high school	1,556	33.0%
		5 He has graduated from university	1,210	26.0%
		6 He has completed master studies or PhD	294	6.2%
		7 Illiterate	7	0.2%
		8 I don't know	401	8.6%
Q7	GRR7	What level of education did your mother (stepmother or father's partner) complete?		
		. Missing	20	.
		1 She's never gone to school	25	0.6%
		2 She has graduated from elementary school	256	5.8%

Original Question	Analysis Variable	Question Code and Label	Unweighted Frequency	Weighted Percent
		3 She has graduated from junior high school	604	14.0%
		4 She has graduated from high school	1,649	35.4%
		5 She has graduated from university	1,379	29.6%
		6 She has completed master studies or PhD	386	8.1%
		7 Illiterate	6	0.1%
		8 I don't know	293	6.4%
Q8	CR5	Have you ever tried or experimented with cigarette smoking, even one or two puffs?		
		. Missing	75	.
		1 Yes	1,380	30.4%
		2 No	3,163	69.6%
Q9	CR6	How old were you when you first tried to smoke?		
		. Missing	70	.
		1 I have never tried smoking a cigarette	3,220	70.9%
		2 7 years old or younger	141	3.2%
		3 8 or 9 years old	92	2.1%
		4 10 or 11 years old	175	4.0%
		5 12 or 13 years old	501	10.9%
		6 14 or 15 years old	410	8.6%
		7 16 years old or older	9	0.2%
Q10	CR7	During the past 30 days, on how many days did you smoke cigarettes?		
		. Missing	125	.
		1 0 days	3,999	89.1%
		2 1 or 2 days	146	3.2%
		3 3 to 5 days	53	1.2%
		4 6 to 9 days	53	1.2%
		5 10 to 19 days	62	1.3%
		6 20 to 29 days	40	0.9%
		7 All 30 days	140	3.1%
Q11	CR8	Please think about the days you smoked cigarettes during the past month (30 days). How many cigarettes did you usually smoke per day?		
		. Missing	170	.
		1 I did not smoke cigarettes during the past 30 days	3,945	88.8%
		2 Less than 1 cigarette per day	111	2.5%
		3 1 cigarette per day	93	2.1%
		4 2 to 5 cigarettes per day	131	2.8%
		5 6 to 10 cigarettes per day	83	1.7%
		6 11 to 20 cigarettes per day	47	1.1%
		7 More than 20 cigarettes per day	38	0.9%
Q12	OR5	During the past 30 days, how often did you smoke hand-rolled cigarettes?		
		. Missing	113	.
		1 I did not smoke hand-rolled cigarettes during the past 30 days	4,103	91.1%
		2 Less than once a week	155	3.5%
		3 At least once a week but not every day	142	3.2%
		4 Every day	105	2.3%
Q13	CR9	Have you ever tried or experimented with some type of smoking tobacco products other than cigarettes from ordinary cigarettes trade (such as rolling, pipe, cigars, cigarillos, hookah)?		
		. Missing	175	.
		1 Yes	778	17.1%
		2 No	3,665	82.9%

Original Question	Analysis Variable	Question Code and Label	Unweighted Frequency	Weighted Percent
Q14	CR10	During the past 30 days, did you use any form of smoked tobacco products other than cigarettes (such as rolling, pipe, cigars, cigarillos, hookah)?		
		. Missing	177	.
		1 Yes	375	8.2%
		2 No	4,066	91.8%
Q15	GRR15	Where do you usually smoke? (select only one response)		
		. Missing	40	.
		1 I have never smoked	3,887	85.0%
		2 At home	96	2.2%
		3 At social events	51	1.0%
		4 At friends' houses	63	1.4%
		5 At school during the school hours	44	1.0%
		6 Outside school during the school hours	18	0.4%
		7 In public places (e.g. streets, cafeterias, parks, malls)	277	5.7%
		8 In other places	142	3.3%
Q16	CR11	Do you ever smoke or feel that you want to smoke just when you wake up in the morning?		
		. Missing	18	.
		1 I have never smoked	3,556	77.4%
		2 No, I don't smoke or ever feel that I want to smoke just when I wake up in the morning	783	17.1%
		3 Yes, I sometimes smoke or feel that I want to smoke just when I wake up in the morning	178	3.8%
		4 Yes, I always smoke or feel that I want to smoke just when I wake up in the morning	83	1.7%
Q17	GRR17	How long after smoking a cigarette do you start thinking about the next one?		
		. Missing	25	.
		1 I don't smoke tobacco	3,929	85.7%
		2 I never feel a strong desire to smoke again after smoking tobacco	353	7.7%
		3 Within 60 minutes	162	3.4%
		4 1 to 2 hours	67	1.5%
		5 More than 2 hours to 4 hours	24	0.5%
		6 More than 4 hours but less than one full day	17	0.3%
		7 1 to 3 days	17	0.4%
		8 4 days or more	24	0.5%
Q18	CR13	Have you ever tried or experimented with some type of tobacco product in any way other than smoking (such as inhaled smoke, chew tobacco or tobacco candy)?		
		. Missing	66	.
		1 Yes	142	3.5%
		2 No	4,410	96.5%
Q19	CR14	During the past month (30 days), did you use some type of tobacco product in any way other than smoking (such as inhaled smoke, chew tobacco or tobacco candy)?		
		. Missing	68	.
		1 Yes	63	1.5%
		2 No	4,487	98.5%
Q20	GRR20	Have you ever tried or experimented with the electronic cigarette?		
		. Missing	43	.
		1 Yes	579	12.3%
		2 No	3,996	87.7%
Q21	GRR21	During the past 30 days, have you used the electronic cigarette?		
		. Missing	58	.

Original Question	Analysis Variable	Question Code and Label	Unweighted Frequency	Weighted Percent
		1 Yes	125	2.9%
		2 No	4,435	97.1%
Q22	GRR22	In your family does anyone use the electronic cigarette?		
		. Missing	51	.
		1 Yes	576	12.1%
		2 No	3,991	87.9%
Q23	CR15	Do you want to stop smoking now?		
		. Missing	33	.
		1 I have never smoked	3,524	76.8%
		2 I don't smoke now	690	15.3%
		3 Yes	170	3.5%
		4 No	201	4.4%
Q24	CR16	During the last year (12 months), have you ever tried to quit smoking?		
		. Missing	41	.
		1 I have never smoked	3,581	78.3%
		2 I did not smoke during the past 12 months	477	10.6%
		3 Yes	325	6.9%
		4 No	194	4.3%
Q25	CR17	Do you think you would be able to quit smoking if you wanted?		
		. Missing	27	.
		1 I don't smoke tobacco	3,557	77.5%
		2 I don't smoke now	511	11.3%
		3 Yes	444	9.5%
		4 No	79	1.7%
Q26	CR18	Have you ever received help or advice to quit smoking? (select only one response)		
		. Missing	35	.
		1 I have never smoked	3,683	80.2%
		2 Yes, from a program or health professional	36	0.9%
		3 Yes, from a friend	257	5.5%
		4 Yes, from a family member	122	2.5%
		5 Yes, all of the above (from a program or healthcare professional, friends or family members)	67	1.5%
		6 No	418	9.4%
Q27	GRR27	How long ago did you quit smoking?		
		. Missing	40	.
		1 I have never smoked	3,719	81.0%
		2 I have not quit smoking	290	6.3%
		3 Less than a month	123	2.7%
		4 1-3 months	128	2.7%
		5 4-11 months	94	2.2%
		6 One year	100	2.2%
		7 2 years	57	1.4%
		8 3 years or more	67	1.5%
Q28	GRR28	What was the main reason you decided to quit smoking? (select one response only)		
		. Missing	27	.
		1 I have never smoked	3,698	80.4%
		2 I have not quit smoking	256	5.5%
		3 To improve my health	332	7.4%
		4 To save money	24	0.6%

Original Question	Analysis Variable	Question Code and Label	Unweighted Frequency	Weighted Percent
		5 Because my family does not like it	34	0.8%
		6 Because my friends do not like it	16	0.4%
		7 Because my hair, clothes and breath smell bad	51	1.1%
		8 An other reason	180	3.9%
Q29	CR19	During the past 7 days, on how many days has anyone smoked inside your home, in your presence?		
		. Missing	31	.
		1 0 days	2,013	43.8%
		2 1 to 2 days	724	16.1%
		3 3 to 4 days	405	9.1%
		4 5 to 6 days	263	5.6%
		5 7 days	1,182	25.4%
Q30	GRR30	Who smokes in the house?		
		. Missing	27	.
		1 Father	320	6.3%
		2 Mother	1,301	27.8%
		3 Brothers	863	19.1%
		4 Both the parents	2,106	46.8%
		5 More than 2 persons	1	0.0%
Q31	GRR31	How many cigarettes on average do your parents smoke inside your own home a day?		
		. Missing	30	.
		1 0	271	5.4%
		2 1 to 5	1,032	21.5%
		3 6 to 10	795	17.4%
		4 11 to 20	2,486	55.6%
		5 More than 20	4	0.1%
Q32	CR20	During the past 7 days, on how many days has anyone smoked in your presence, inside any enclosed public place, other than your home (such as school, shops, restaurants, shopping malls, movie theaters)?		
		. Missing	42	.
		1 0 days	1,477	32.7%
		2 1 to 2 days	1,248	27.1%
		3 3 to 4 days	692	15.0%
		4 5 to 6 days	340	7.2%
		5 7 days	819	17.9%
Q33	CR21	During the past 7 days, on how many days has anyone smoked in your presence, at any outdoor public place (such as playgrounds, sidewalks, entrances to buildings, parks, beaches)?		
		. Missing	30	.
		1 0 days	1,425	31.9%
		2 1 to 2 days	1,197	26.0%
		3 3 to 4 days	690	14.9%
		4 5 to 6 days	371	7.8%
		5 7 days	905	19.4%
Q34	CR22	During the past 30 days, did you see any one smoke inside the school building or outside on school property?		
		. Missing	39	.
		1 Yes	3,508	76.0%
		2 No	1,071	24.0%
Q35	CR23	Do you think the smoke from other people's tobacco smoking is harmful to you?		
		. Missing	22	.

Original Question	Analysis Variable	Question Code and Label	Unweighted Frequency	Weighted Percent
		1 Definitely not	145	3.4%
		2 Probably not	125	2.9%
		3 Probably yes	773	16.6%
		4 Definitely yes	3,553	77.1%
Q36	CR24	Are you in favor of banning smoking inside enclosed public places (such as schools, shops, restaurants, shopping malls, movie theaters)?		
		. Missing	52	.
		1 Yes	3,751	81.9%
		2 No	815	18.1%
Q37	CR25	Are you in favor of banning smoking at outdoor public places (such as playgrounds, sidewalks, entrances to buildings, parks, beaches)?		
		. Missing	49	.
		1 Yes	2,573	57.0%
		2 No	1,996	43.0%
Q38	GRR38	Do you know if in Greece there is a law that bans smoking in school and public places?		
		. Missing	36	.
		1 Yes	2,563	55.5%
		2 No	302	6.9%
		3 I don't know	1,717	37.6%
Q39	CR26	The last time you smoked cigarettes during the past 30 days, how did you get them? (select only one response)		
		. Missing	120	.
		1 I did not smoke any cigarettes during the past 30 days	3,933	87.7%
		2 I bought them in a store or shop	69	1.7%
		3 I bought them from a street vendor	20	0.4%
		4 I bought them at a kiosk	281	5.9%
		5 I bought them from a vending machine	9	0.2%
		6 I got them from someone else	146	3.2%
		7 I got them some other way	40	0.9%
Q40	CR27	During the past month (30 days), did anyone refuse to sell you cigarettes because of your age?		
		. Missing	104	.
		1 I did not try to buy cigarettes during the past 30 days	3,915	86.8%
		2 Yes, someone refused to sell me cigarettes because of my age	88	2.0%
		3 No, my age did not keep me from buying cigarettes	511	11.3%
Q41	OR22	Can you purchase cigarettes or other tobacco products near your school?		
		. Missing	65	.
		1 Yes	1,834	39.5%
		2 No	1,110	25.0%
		3 I don't know	1,609	35.5%
Q42	CR28	The last time you bought cigarettes during the past month (30 days), how did you buy them?		
		. Missing	99	.
		1 I did not buy cigarettes during the past 30 days	3,934	87.3%
		2 I bought them in a pack	397	8.7%
		3 I bought individual sticks (singles)	26	0.6%
		4 I bought them in a carton	19	0.4%
		5 I bought them in rolls	95	2.1%
		6 I bought tobacco and rolled my own	48	1.0%

Original Question	Analysis Variable	Question Code and Label	Unweighted Frequency	Weighted Percent
Q43	GRR43	On average, how much do you think a pack of 20 cigarettes costs?		
		. Missing	50	.
		1 2,00 to 3,00 €	463	10.5%
		2 3,00 to 4,00 €	2,150	47.4%
		3 4,00 to 5,00 €	577	12.6%
		4 5,00 to 6,00 €	111	2.1%
		5 6,00 to 7,00 €	75	1.5%
		6 I don't know	1,192	25.8%
Q44	GRR44	During the past week, how much money did you spend on buying cigarettes or tobacco?		
		. Missing	91	.
		1 I don't smoke	3,316	74.8%
		2 I don't buy cigarettes or tobacco	851	17.4%
		3 Up to 5 €	175	3.7%
		4 6 to 10 €	97	2.2%
		5 11 to 20 €	35	0.8%
		6 21 to 25 €	16	0.3%
		7 26 to 30 €	10	0.2%
		8 More than 30 €	27	0.6%
Q45	CR30	During the past month (30 days), did you see or hear any anti-tobacco messages on television, radio, internet, billboards, posters, newspapers, magazines, or movies?		
		. Missing	81	.
		1 Yes	2,424	53.1%
		2 No	2,113	46.9%
Q46	CR31	During the past month (30 days), did you see or hear any anti-tobacco messages at sports events, fairs, concerts, or community events, or social gatherings?		
		. Missing	61	.
		1 I did not go to sports events, fairs, concerts, or community events, or social gatherings in the past 30 days	1,129	25.1%
		2 Yes	1,049	23.3%
		3 No	2,379	51.6%
Q47	CR32	During the past month (30 days), did you see any health warnings on cigarette packages?		
		. Missing	117	.
		1 Yes, but I didn't think much of them	1,271	27.7%
		2 Yes, and they led me to think about quitting smoking or not starting smoking	1,622	36.4%
		3 No	1,608	35.9%
Q48	OR25	During the past month (30 days), did you see any signs prohibiting the sale of cigarettes and tobacco to minors under 18 years?		
		. Missing	100	.
		1 Yes	1,290	29.0%
		2 No	3,228	71.0%
Q49	CR33	During the past 12 months, were you taught in any of your classes about the dangers of tobacco use?		
		. Missing	62	.
		1 Yes	2,801	60.5%
		2 No	1,361	30.5%
		3 I don't know	394	9.0%
Q50	CR34	During the past 30 days, did you see any people using tobacco when you watched TV, videos, or movies?		
		. Missing	43	.
		1 I did not watch TV, videos, or movies in the past 30 days	262	6.4%

Original Question	Analysis Variable	Question Code and Label	Unweighted Frequency	Weighted Percent
		2 Yes	3,807	82.3%
		3 No	506	11.3%
Q51	CR35	During the past 30 days, did you see any advertisements or promotions for tobacco products at points of sale (such as stores, shops, kiosks, etc.)?		
		. Missing	75	.
		1 I did not visit any points of sale in the past 30 days	1,157	25.8%
		2 Yes	1,391	30.0%
		3 No	1,995	44.1%
Q52	GRR52	Would you ever use or wear something that has a tobacco company or tobacco product name or picture on it such as a lighter, t-shirt, hat, or sunglasses?		
		. Missing	62	.
		1 Yes	543	11.9%
		2 No	3,220	70.7%
		3 Maybe	793	17.4%
Q53	CR37	Do you have something (for example, t-shirt, pen, backpack) with a tobacco product brand logo on it?		
		. Missing	68	.
		1 Yes	441	9.5%
		2 No	4,109	90.5%
Q54	CR38	Has a person working for a tobacco company ever offered you a free tobacco product?		
		. Missing	68	.
		1 Yes	265	5.8%
		2 No	4,285	94.2%
Q55	CR39	If one of your best friends offered you a tobacco product, would you use it?		
		. Missing	54	.
		1 Definitely not	3,423	75.3%
		2 Probably not	565	12.5%
		3 Probably yes	379	8.0%
		4 Definitely yes	197	4.2%
Q56	CR40	At anytime during the next 12 months do you think you will use any form of tobacco?		
		. Missing	48	.
		1 Definitely not	3,471	76.3%
		2 Probably not	450	9.8%
		3 Probably yes	348	7.5%
		4 Definitely yes	301	6.4%
Q57	CR41	Once someone has started smoking tobacco, do you think it would be difficult for them to quit?		
		. Missing	66	.
		1 Definitely not	449	10.2%
		2 Probably not	378	8.3%
		3 Probably yes	1,838	39.7%
		4 Definitely yes	1,887	41.8%
Q58	CR42	Do you think smoking tobacco helps people feel more comfortable or less comfortable at celebrations, parties, or in other social gatherings?		
		. Missing	143	.
		1 More comfortable	1,142	25.6%
		2 Less comfortable	1,307	29.1%
		3 No difference whether smoking or not	2,026	45.3%

Original Question	Analysis Variable	Question Code and Label	Unweighted Frequency	Weighted Percent
Q59	CR43	Do you agree or disagree with the following: "I think I might enjoy smoking a cigarette."		
		. Missing	71	.
		1 I currently smoke cigarettes	185	4.1%
		2 Strongly agree	108	2.6%
		3 Agree	295	6.3%
		4 Disagree	1,154	25.0%
		5 Strongly disagree	2,805	61.9%
Q60	GRR60	In your opinion, are hand-rolled cigarettes less harmful than regular cigarettes?		
		. Missing	194	.
		1 I totally agree	551	12.9%
		2 I agree	1,621	36.6%
		3 I disagree	1,409	31.6%
		4 I totally disagree	843	19.0%
Q61	SR1	Have you ever tried or experimented with shisha, even one or two puffs?		
		. Missing	98	.
		1 Yes	782	16.4%
		2 No	3,738	83.6%
Q62	GRR62	The last time you did shisha during the past 30 days, where did you? (select only one response)		
		. Missing	142	.
		1 I did not smoke shisha during the past 30 days	3,901	87.8%
		2 At home	203	4.6%
		3 At a café	158	3.0%
		4 At a restaurant or tavern	12	0.3%
		5 At a bar or club or nightclub	60	1.3%
		6 Other	142	3.1%
