# Supplementary material for: Prevalence of Electronic Cigarette Use and Its Determinants among 13-to-15-Year-Old Students in Greece: Results from the 2013 Global Youth Tobacco Survey (GYTS)
Source: Int J Environ Res Public Health. 2020 Mar 4;17(5):1671. doi: 10.3390/ijerph17051671 (PMC7084902; doi:10.3390/ijerph17051671)
Supplement: Supplementary file 1 [file ijerph-17-01671-s001.zip › ijerph-732136-supplementary/File S3--Output of statistical analysis.pdf]

Frequencies

Statistics

		Age	Gender	Pocket money	Father's education	Mother's education	Ever tobacco use	Tobacco smoking	Ever e-cig use	E-cigarette use	Family e-cig use	Family smoking	Smoking intention	Ever shisha use
N	Valid	4096	4095	4084	3749	3837	4034	3991	4066	4057	4058	4096	4057	4030
	Missing	0	1	12	347	259	62	105	30	39	38	0	39	66

Frequency Table

Age

		Frequency	Percent	Valid Percent	Cumulative Percent
Valid	13 years old	1542	37,7	37,7	37,7
	14 years old	1432	35,0	35,0	72,6
	15 years old	1122	27,4	27,4	100,0
	Total	4096	100,0	100,0	

Gender

		Frequency	Percent	Valid Percent	Cumulative Percent
Valid	Male	2108	51,5	51,5	51,5
	Female	1987	48,5	48,5	100,0
	Total	4095	100,0	100,0	
Missing	System	1	,0		
Total		4096	100,0		

Pocket money

		Frequency	Percent	Valid Percent	Cumulative Percent
Valid	No money	241	5,9	5,9	5,9
	1-9€	2245	54,8	55,0	60,9
	10-19€	1162	28,4	28,5	89,3

		Frequency	Percent	Valid Percent	Cumulative Percent
	>20€	436	10,7	10,7	100,0
	Total	4084	99,7	100,0	
Missing	System	12	,3		
Total		4096	100,0		

Father's education

		Frequency	Percent	Valid Percent	Cumulative Percent
Valid	High school or above	2707	66,1	72,2	72,2
	Middle school or below	1042	25,5	27,8	100,0
	Total	3749	91,5	100,0	
Missing	System	347	8,5		
Total		4096	100,0		

Mother's education

		Frequency	Percent	Valid Percent	Cumulative Percent
Valid	High school or above	2997	73,2	78,1	78,1
	Middle school or below	840	20,5	21,9	100,0
	Total	3837	93,7	100,0	
Missing	System	259	6,3		
Total		4096	100,0		

Ever tobacco use

		Frequency	Percent	Valid Percent	Cumulative Percent
Valid	Yes	1228	30,0	30,4	30,4
	No	2806	68,5	69,6	100,0
	Total	4034	98,5	100,0	
Missing	System	62	1,5		
Total		4096	100,0		

Tobacco smoking

		Frequency	Percent	Valid Percent	Cumulative Percent
Valid	Yes	402	9,8	10,1	10,1
	No	3588	87,6	89,9	100,0

		Frequency	Percent	Valid Percent	Cumulative Percent
	Total	3991	97,4	100,0	
Missing	System	105	2,6		
Total		4096	100,0		

Ever e-cig use

		Frequency	Percent	Valid Percent	Cumulative Percent
Valid	Yes	498	12,2	12,3	12,3
	No	3568	87,1	87,7	100,0
	Total	4066	99,3	100,0	
Missing	System	30	,7		
Total		4096	100,0		

E-cigarette use

		Frequency	Percent	Valid Percent	Cumulative Percent
Valid	Yes	115	2,8	2,8	2,8
	No	3942	96,2	97,2	100,0
	Total	4057	99,0	100,0	
Missing	System	39	1,0		
Total		4096	100,0		

Family e-cig use

		Frequency	Percent	Valid Percent	Cumulative Percent
Valid	Yes	498	12,2	12,3	12,3
	No	3560	86,9	87,7	100,0
	Total	4058	99,1	100,0	
Missing	System	38	,9		
Total		4096	100,0		

Family smoking

		Frequency	Percent	Valid Percent	Cumulative Percent
Valid	Someone	4077	99,5	99,5	99,5
	No one	19	,5	,5	100,0
	Total	4096	100,0	100,0	

Frequency Table

Susceptibility to smoking?

		Frequency	Percent	Valid Percent	Cumulative Percent
Valid	1	323	12,8	12,9	12,9
	2	2187	86,5	87,1	100,0
	Total	2510	99,3	100,0	
Missing	System	17	,7		
Total		2527	100,0		

T-Test

One-Sample Statistics

	N	Mean	Std. Deviation	Std. Error Mean
Gender	4095	1,49	,500	,008
Father's education	3749	1,28	,448	,007
Mother's education	3837	1,22	,414	,007
Ever tobacco use	4034	1,70	,460	,007
Tobacco smoking	3991	1,90	,301	,005
Ever e-cig use	4066	1,88	,328	,005
E-cigarette use	4057	1,97	,166	,003
Family e-cig use	4058	1,88	,328	,005
Family smoking	4096	1,00	,068	,001
Susceptibility to smoking?	4058	1,70	,460	,007
Ever shisha use	4030	1,84	,370	,006

One-Sample Test

Test Value = 1

	t	df	Sig. (2-tailed)	Mean Difference	95% Confidence Interval of the Difference	
					Lower	Upper
Gender	62,111	4094	,000	,485	,47	,50
Father's education	37,994	3748	,000	,278	,26	,29
Mother's education	32,781	3836	,000	,219	,21	,23
Ever tobacco use	95,999	4033	,000	,696	,68	,71
Tobacco smoking	188,611	3990	,000	,899	,89	,91
Ever e-cig use	170,629	4065	,000	,877	,87	,89
E-cigarette use	373,170	4056	,000	,972	,97	,98
Family e-cig use	170,267	4057	,000	,877	,87	,89
Family smoking	4,389	4095	,000	,005	,00	,01
Susceptibility to smoking?	96,321	4057	,000	,696	,68	,71
Ever shisha use	143,417	4029	,000	,836	,82	,85

T-Test

One-Sample Statistics

	N	Mean	Std. Deviation	Std. Error Mean
Susceptibility to smoking?	2510	1,87	,335	,007

One-Sample Test

	Test Value = 1					
	t	df	Sig. (2-tailed)	Mean Difference	95% Confidence Interval of the Difference	
					Lower	Upper
Susceptibility to smoking?	130,325	2509	,000	,871	,86	,88

Frequencies

Statistics

		Ever tobacco use	Tobacco smoking	Ever other smoked tobacco product	Other smoked tobacco product	Ever other non-smoke tobacco	Non-smoked tobacco product	Ever e-cig use	E-cigarette use	Any combustible product (Ever)	Any nicotine-containing product (Ever)	Any combustible product (Current)	Any nicotine-containing product (Current)
N	Valid	4034	3991	3960	3951	4045	4048	4066	4057	3974	3976	3900	3883
	Missing	62	105	136	145	51	48	30	39	122	120	196	213

Frequency Table

Ever tobacco use

		Frequency	Percent	Valid Percent	Cumulative Percent
Valid	Yes	1228	30,0	30,4	30,4
	No	2806	68,5	69,6	100,0
	Total	4034	98,5	100,0	
Missing	System	62	1,5		
Total		4096	100,0		

Tobacco smoking

		Frequency	Percent	Valid Percent	Cumulative Percent
Valid	Yes	402	9,8	10,1	10,1
	No	3588	87,6	89,9	100,0
	Total	3991	97,4	100,0	
Missing	System	105	2,6		
Total		4096	100,0		

Ever other smoked tobacco product

		Frequency	Percent	Valid Percent	Cumulative Percent

		Frequency	Percent	Valid Percent	Cumulative Percent
Valid	Yes	663	16,2	16,7	16,7
	No	3296	80,5	83,3	100,0
	Total	3960	96,7	100,0	
Missing	System	136	3,3		
Total		4096	100,0		

Other smoked tobacco product

		Frequency	Percent	Valid Percent	Cumulative Percent
Valid	Yes	301	7,3	7,6	7,6
	No	3650	89,1	92,4	100,0
	Total	3951	96,5	100,0	
Missing	System	145	3,5		
Total		4096	100,0		

Ever other non-smoke tobacco

		Frequency	Percent	Valid Percent	Cumulative Percent
Valid	Yes	137	3,3	3,4	3,4
	No	3908	95,4	96,6	100,0
	Total	4045	98,8	100,0	
Missing	System	51	1,2		
Total		4096	100,0		

Non-smoked tobacco product

		Frequency	Percent	Valid Percent	Cumulative Percent
Valid	Yes	61	1,5	1,5	1,5
	No	3987	97,3	98,5	100,0
	Total	4048	98,8	100,0	
Missing	System	48	1,2		
Total		4096	100,0		

Ever e-cig use

		Frequency	Percent	Valid Percent	Cumulative Percent
Valid	Yes	498	12,2	12,3	12,3

		Frequency	Percent	Valid Percent	Cumulative Percent
	No	3568	87,1	87,7	100,0
	Total	4066	99,3	100,0	
Missing	System	30	,7		
Total		4096	100,0		

E-cigarette use

		Frequency	Percent	Valid Percent	Cumulative Percent
Valid	Yes	115	2,8	2,8	2,8
	No	3942	96,2	97,2	100,0
	Total	4057	99,0	100,0	
Missing	System	39	1,0		
Total		4096	100,0		

Any combustible product (Ever)

		Frequency	Percent	Valid Percent	Cumulative Percent
Valid	1	1386	33,8	34,9	34,9
	2	2588	63,2	65,1	100,0
	Total	3974	97,0	100,0	
Missing	System	122	3,0		
Total		4096	100,0		

Any nicotine-containing product (Ever)

		Frequency	Percent	Valid Percent	Cumulative Percent
Valid	1	1511	36,9	38,0	38,0
	2	2464	60,2	62,0	100,0
	Total	3976	97,1	100,0	
Missing	System	120	2,9		
Total		4096	100,0		

Any combustible product (Current)

		Frequency	Percent	Valid Percent	Cumulative Percent
Valid	Yes	518	12,6	13,3	13,3
	No	3382	82,6	86,7	100,0

	Total	3900	95,2	100,0	
Missing	System	196	4,8		
Total		4096	100,0		

Any nicotine-containing product (Current)

		Frequency	Percent	Valid Percent	Cumulative Percent
Valid	Yes	592	14,4	15,2	15,2
	No	3291	80,3	84,8	100,0
	Total	3883	94,8	100,0	
Missing	System	213	5,2		
Total		4096	100,0		

Crosstabs

Case Processing Summary

	Cases					
	Valid		Missing		Total	
	N	Percent	N	Percent	N	Percent
Age * Tobacco smoking	3990	97,4%	106,000	2,6%	4096	100,0%
Age * Other smoked tobacco product	3952[a]	96,5%	144,000	3,5%	4096	100,0%
Age * Non-smoked tobacco product	4048[a]	98,8%	48,000	1,2%	4096	100,0%
Age * E-cigarette use	4057[a]	99,0%	39,000	1,0%	4096	100,0%
Age * Any combustible product (Current)	3901[a]	95,2%	195,000	4,8%	4096	100,0%
Age * Any nicotine-containing product (Current)	3882[a]	94,8%	214,000	5,2%	4096	100,0%

a. Number of valid cases is different from the total count in the crosstabulation table because the cell counts have been rounded.

Age * Tobacco smoking Crosstabulation

			Tobacco smoking		
			Yes	No	Total
Age	13 years old	Count	71	1437	1508

		% within Age	4,7%	95,3%	100,0%
	14 years old	Count	147	1247	1394
		% within Age	10,5%	89,5%	100,0%
	15 years old	Count	184	904	1088
		% within Age	16,9%	83,1%	100,0%
Total		Count	402	3588	3990
		% within Age	10,1%	89,9%	100,0%

Age * Other smoked tobacco product Crosstabulation

			Other smoked tobacco product		Total
			Yes	No	
Age	13 years old	Count	41	1443	1484
		% within Age	2,8%	97,2%	100,0%
	14 years old	Count	119	1263	1382
		% within Age	8,6%	91,4%	100,0%
	15 years old	Count	141	945	1086
		% within Age	13,0%	87,0%	100,0%
Total		Count	301	3651	3952
		% within Age	7,6%	92,4%	100,0%

Age * Non-smoked tobacco product Crosstabulation

			Non-smoked tobacco product		Total
			Yes	No	
Age	13 years old	Count	15	1502	1517
		% within Age	1,0%	99,0%	100,0%
	14 years old	Count	23	1395	1418
		% within Age	1,6%	98,4%	100,0%
	15 years old	Count	23	1090	1113
		% within Age	2,1%	97,9%	100,0%
Total		Count	61	3987	4048
		% within Age	1,5%	98,5%	100,0%

Age * E-cigarette use Crosstabulation

			E-cigarette use	Total

			Yes	No	
Age	13 years old	Count	17	1502	1519
		% within Age	1,1%	98,9%	100,0%
	14 years old	Count	46	1377	1423
		% within Age	3,2%	96,8%	100,0%
	15 years old	Count	52	1063	1115
		% within Age	4,7%	95,3%	100,0%
Total		Count	115	3942	4057
		% within Age	2,8%	97,2%	100,0%

Age * Any combustible product (Current) Crosstabulation

			Any combustible product (Current)		
			Yes	No	Total
Age	13 years old	Count	83	1379	1462
		% within Age	5,7%	94,3%	100,0%
	14 years old	Count	193	1167	1360
		% within Age	14,2%	85,8%	100,0%
	15 years old	Count	242	837	1079
		% within Age	22,4%	77,6%	100,0%
Total		Count	518	3383	3901
		% within Age	13,3%	86,7%	100,0%

Age * Any nicotine-containing product (Current) Crosstabulation

			Any nicotine-containing product (Current)		
			Yes	No	Total
Age	13 years old	Count	105	1335	1440
		% within Age	7,3%	92,7%	100,0%
	14 years old	Count	223	1143	1366
		% within Age	16,3%	83,7%	100,0%
	15 years old	Count	263	813	1076
		% within Age	24,4%	75,6%	100,0%
Total		Count	591	3291	3882
		% within Age	15,2%	84,8%	100,0%

Crosstabs

Case Processing Summary

	Cases					
	Valid		Missing		Total	
	N	Percent	N	Percent	N	Percent
Age * E-cigarette use	4057	99,0%	39,000	1,0%	4096	100,0%
Gender * E-cigarette use	4056[a]	99,0%	40,000	1,0%	4096	100,0%
Pocket money * E-cigarette use	4047[a]	98,8%	49,000	1,2%	4096	100,0%
Father's education * E-cigarette use	3715[a]	90,7%	381,000	9,3%	4096	100,0%
Mother's education * E-cigarette use	3803[a]	92,8%	293,000	7,2%	4096	100,0%
Family e-cig use * E-cigarette use	4032[a]	98,4%	64,000	1,6%	4096	100,0%
Family smoking * E-cigarette use	4055[a]	99,0%	41,000	1,0%	4096	100,0%
Ever shisha use * E-cigarette use	3995[a]	97,5%	101,000	2,5%	4096	100,0%
Tobacco smoking * E-cigarette use	3958[a]	96,6%	138,000	3,4%	4096	100,0%

a. Number of valid cases is different from the total count in the crosstabulation table because the cell counts have been rounded.

Crosstab

			E-cigarette use		
			Yes	No	Total
Age	13 years old	Count	17	1502	1519
		% within Age	1,1%	98,9%	100,0%
	14 years old	Count	46	1377	1423
		% within Age	3,2%	96,8%	100,0%
	15 years old	Count	52	1063	1115
		% within Age	4,7%	95,3%	100,0%
Total		Count	115	3942	4057

	% within Age	2,8%	97,2%	100,0%

Chi-Square Tests

	Value	df	Asymptotic Significance (2-sided)
Pearson Chi-Square	30,592[a]	2	,000
Likelihood Ratio	33,139	2	,000
Linear-by-Linear Association	30,197	1	,000
N of Valid Cases	4057		

a. 0 cells (,0%) have expected count less than 5. The minimum expected count is 31,61.

Risk Estimate

	Value
Odds Ratio for Age (13 years old / 14 years old)	[a]

a. Risk Estimate statistics cannot be computed. They are only computed for a 2*2 table without empty cells.

Gender * E-cigarette use

Crosstab

			E-cigarette use		
			Yes	No	Total
Gender	Male	Count	82	2004	2086
		% within Gender	3,9%	96,1%	100,0%
	Female	Count	33	1937	1970
		% within Gender	1,7%	98,3%	100,0%
Total		Count	115	3941	4056
		% within Gender	2,8%	97,2%	100,0%

Chi-Square Tests

	Value	df	Asymptotic Significance (2-sided)	Exact Sig. (2-sided)	Exact Sig. (1-sided)
Pearson Chi-Square	18,715[a]	1	,000		
Continuity Correction[b]	17,905	1	,000		
Likelihood Ratio	19,382	1	,000		
Fisher's Exact Test				,000	,000
Linear-by-Linear Association	18,710	1	,000		
N of Valid Cases	4056				

a. 0 cells (,0%) have expected count less than 5. The minimum expected count is 55,86.

b. Computed only for a 2x2 table

Risk Estimate

	Value	95% Confidence Interval	
		Lower	Upper
Odds Ratio for Gender (Male / Female)	2,402	1,596	3,615
For cohort E-cigarette use = Yes	2,347	1,574	3,498
For cohort E-cigarette use = No	,977	,967	,987
N of Valid Cases	4056		

Pocket money * E-cigarette use

Crosstab

			E-cigarette use		
			Yes	No	Total
Pocket money	No money	Count	5	231	236
		% within Pocket money	2,1%	97,9%	100,0%
	1-9€	Count	52	2170	2222
		% within Pocket money	2,3%	97,7%	100,0%

	10-19€	Count	38	1117	1155
		% within Pocket money	3,3%	96,7%	100,0%
	>20€	Count	17	417	434
		% within Pocket money	3,9%	96,1%	100,0%
Total		Count	112	3935	4047
		% within Pocket money	2,8%	97,2%	100,0%

Chi-Square Tests

	Value	df	Asymptotic Significance (2-sided)
Pearson Chi-Square	5,180[a]	3	,159
Likelihood Ratio	4,989	3	,173
Linear-by-Linear Association	4,892	1	,027
N of Valid Cases	4047		

a. 0 cells (,0%) have expected count less than 5. The minimum expected count is 6,53.

Risk Estimate

	Value
Odds Ratio for Pocket money (No money / 1-9€)	[a]

a. Risk Estimate statistics cannot be computed. They are only computed for a 2*2 table without empty cells.

Father's education * E-cigarette use

Crosstab

			E-cigarette use		
			Yes	No	Total
Father's education	High school or above	Count	66	2618	2684
		% within Father's education	2,5%	97,5%	100,0%

	Middle school or below	Count	39	992	1031
		% within Father's education	3,8%	96,2%	100,0%
Total		Count	105	3610	3715
		% within Father's education	2,8%	97,2%	100,0%

Chi-Square Tests

	Value	df	Asymptotic Significance (2-sided)	Exact Sig. (2-sided)	Exact Sig. (1-sided)
Pearson Chi-Square	4,752[a]	1	,029		
Continuity Correction[b]	4,282	1	,039		
Likelihood Ratio	4,489	1	,034		
Fisher's Exact Test				,035	,021
Linear-by-Linear Association	4,751	1	,029		
N of Valid Cases	3715				

a. 0 cells (,0%) have expected count less than 5. The minimum expected count is 29,14.

b. Computed only for a 2x2 table

Risk Estimate

	Value	95% Confidence Interval Lower	Upper
Odds Ratio for Father's education (High school or above / Middle school or below)	,641	,429	,959
For cohort E-cigarette use = Yes	,650	,440	,959
For cohort E-cigarette use = No	1,014	1,000	1,028
N of Valid Cases	3715		

Mother's education * E-cigarette use

Crosstab

	E-cigarette use	Total

			Yes	No	
Mother's education	High school or above	Count	80	2894	2974
		% within Mother's education	2,7%	97,3%	100,0%
	Middle school or below	Count	31	798	829
		% within Mother's education	3,7%	96,3%	100,0%
Total		Count	111	3692	3803
		% within Mother's education	2,9%	97,1%	100,0%

Chi-Square Tests

	Value	df	Asymptotic Significance (2-sided)	Exact Sig. (2-sided)	Exact Sig. (1-sided)
Pearson Chi-Square	2,520[a]	1	,112		
Continuity Correction[b]	2,163	1	,141		
Likelihood Ratio	2,377	1	,123		
Fisher's Exact Test				,128	,073
Linear-by-Linear Association	2,519	1	,112		
N of Valid Cases	3803				

a. 0 cells (,0%) have expected count less than 5. The minimum expected count is 24,20.

b. Computed only for a 2x2 table

Risk Estimate

	Value	95% Confidence Interval Lower	Upper
Odds Ratio for Mother's education (High school or above / Middle school or below)	,712	,467	1,085
For cohort E-cigarette use = Yes	,719	,479	1,081
For cohort E-cigarette use = No	1,011	,996	1,026
N of Valid Cases	3803		

Family e-cig use * E-cigarette use

Crosstab

			E-cigarette use		
			Yes	No	Total
Family e-cig use	Yes	Count	50	442	492
		% within Family e-cig use	10,2%	89,8%	100,0%
	No	Count	61	3479	3540
		% within Family e-cig use	1,7%	98,3%	100,0%
Total		Count	111	3921	4032
		% within Family e-cig use	2,8%	97,2%	100,0%

Chi-Square Tests

	Value	df	Asymptotic Significance (2-sided)	Exact Sig. (2-sided)	Exact Sig. (1-sided)
Pearson Chi-Square	114,920[a]	1	,000		
Continuity Correction[b]	111,789	1	,000		
Likelihood Ratio	76,679	1	,000		
Fisher's Exact Test				,000	,000
Linear-by-Linear Association	114,892	1	,000		
N of Valid Cases	4032				

a. 0 cells (,0%) have expected count less than 5. The minimum expected count is 13,54.

b. Computed only for a 2x2 table

Risk Estimate

	Value	95% Confidence Interval	
		Lower	Upper
Odds Ratio for Family e-cig use (Yes / No)	6,452	4,382	9,498
For cohort E-cigarette use = Yes	5,898	4,107	8,469
For cohort E-cigarette use = No	,914	,887	,942
N of Valid Cases	4032		

Family smoking * E-cigarette use

Crosstab

			E-cigarette use		
			Yes	No	Total
Family smoking	Someone	Count	112	3929	4041
		% within Family smoking	2,8%	97,2%	100,0%
	No one	Count	2	12	14
		% within Family smoking	14,3%	85,7%	100,0%
Total		Count	114	3941	4055
		% within Family smoking	2,8%	97,2%	100,0%

Chi-Square Tests

	Value	df	Asymptotic Significance (2-sided)	Exact Sig. (2-sided)	Exact Sig. (1-sided)
Pearson Chi-Square	6,770[a]	1	,009		
Continuity Correction[b]	3,211	1	,073		
Likelihood Ratio	3,511	1	,061		
Fisher's Exact Test				,057	,057
Linear-by-Linear Association	6,768	1	,009		
N of Valid Cases	4055				

a. 1 cells (25,0%) have expected count less than 5. The minimum expected count is ,39.

b. Computed only for a 2x2 table

Risk Estimate

	Value	95% Confidence Interval	
		Lower	Upper
Odds Ratio for Family smoking (Someone / No one)	,171	,038	,773
For cohort E-cigarette use = Yes	,194	,053	,709
For cohort E-cigarette use = No	1,134	,916	1,405
N of Valid Cases	4055		

Case Processing Summary

	Cases					
	Valid		Missing		Total	
	N	Percent	N	Percent	N	Percent
Any combustible product (Current) * E-cigarette use	3876	94,6%	220,000	5,4%	4096	100,0%

Any combustible product (Current) * E-cigarette use Crosstabulation

			E-cigarette use		
			Yes	No	Total
Any combustible product (Current)	Yes	Count	64	448	512
		% within Any combustible product (Current)	12,5%	87,5%	100,0%
	No	Count	41	3323	3364
		% within Any combustible product (Current)	1,2%	98,8%	100,0%
Total		Count	105	3771	3876
		% within Any combustible product (Current)	2,7%	97,3%	100,0%

Chi-Square Tests

	Value	df	Asymptotic Significance (2-sided)	Exact Sig. (2-sided)	Exact Sig. (1-sided)
Pearson Chi-Square	214,573[a]	1	,000		
Continuity Correction[b]	210,314	1	,000		
Likelihood Ratio	136,225	1	,000		
Fisher's Exact Test				,000	,000
Linear-by-Linear Association	214,518	1	,000		
N of Valid Cases	3876				

a. 0 cells (,0%) have expected count less than 5. The minimum expected count is 13,87.

b. Computed only for a 2x2 table

Risk Estimate

	Value	95% Confidence Interval

		Lower	Upper
Odds Ratio for Any combustible product (Current) (Yes / No)	11,578	7,728	17,347
For cohort E-cigarette use = Yes	10,256	7,008	15,011
For cohort E-cigarette use = No	,886	,857	,915
N of Valid Cases	3876		

Logistic Regression

Case Processing Summary

Unweighted Cases[a]		N	Percent
Selected Cases	Included in Analysis	3523	86,0
	Missing Cases	573	14,0
	Total	4096	100,0
Unselected Cases		0	,0
Total		4096	100,0

a. If weight is in effect, see classification table for the total number of cases.

Dependent Variable Encoding

Original Value	Internal Value
Yes	0
No	1

Categorical Variables Codings

			Parameter coding	
		Frequency	(1)	(2)
Age	13 years old	1272	,000	,000
	14 years old	1229	1,000	,000
	15 years old	1022	,000	1,000
Gender	Male	1773	,000	
	Female	1750	1,000	
Family e-cig use	Yes	446	,000	

	No	3077	1,000	
Any combustible product (Current)	Yes	470	,000	
	No	3053	1,000	
Father's education	High school or above	2601	,000	
	Middle school or below	922	1,000	

Block 0: Beginning Block

Classification Table[a,b]

	Observed		Predicted		
			E-cigarette use		Percentage Correct
			Yes	No	
Step 0	E-cigarette use	Yes	0	95	,0
		No	0	3437	100,0
	Overall Percentage				97,3

a. Constant is included in the model.

b. The cut value is ,500

Variables in the Equation

		B	S.E.	Wald	df	Sig.	Exp(B)
Step 0	Constant	3,588	,104	1190,846	1	,000	36,159

Variables not in the Equation

			Score	df	Sig.
Step 0	Variables	Age	29,051	2	,000
		Age(1)	,160	1	,689
		Age(2)	20,925	1	,000
		Gender(1)	23,184	1	,000
		Father's education(1)	2,435	1	,119
		Any combustible product (Current)(1)	190,153	1	,000
		Family e-cig use(1)	96,512	1	,000

	Overall Statistics	295,546	6	,000

Block 1: Method = Enter

Omnibus Tests of Model Coefficients

		Chi-square	df	Sig.
Step 1	Step	200,663	6	,000
	Block	200,663	6	,000
	Model	200,663	6	,000

Model Summary

Step	-2 Log likelihood	Cox & Snell R Square	Nagelkerke R Square
1	674,236[a]	,055	,252

a. Estimation terminated at iteration number 8 because parameter estimates changed by less than ,001.

Hosmer and Lemeshow Test

Step	Chi-square	df	Sig.
1	6,826	8	,556

Contingency Table for Hosmer and Lemeshow Test

		E-cigarette use = Yes		E-cigarette use = No		
		Observed	Expected	Observed	Expected	Total
Step 1	1	59	56,243	307	309,536	366
	2	16	14,465	328	329,541	344
	3	8	6,665	347	348,335	355
	4	3	3,384	219	218,921	222
	5	2	4,113	330	328,118	332
	6	4	1,957	234	235,871	238
	7	0	1,864	269	266,780	269

	8	2	2,606	417	416,956	420
	9	1	2,413	460	458,937	461
	10	0	1,354	526	524,422	526

Classification Table[a]

Observed			Predicted: E-cigarette use Yes	No	Percentage Correct
Step 1	E-cigarette use	Yes	2	93	2,5
		No	4	3433	99,9
	Overall Percentage				97,3

a. The cut value is ,500

Variables in the Equation

		B	S.E.	Wald	df	Sig.	Exp(B)	95% C.I.for EXP(B) Lower	Upper
Step 1[a]	Age			9,862	2	,007			
	Age(1)	-,696	,341	4,158	1	,041	,499	,255	,973
	Age(2)	-1,053	,338	9,709	1	,002	,349	,180	,677
	Gender(1)	,942	,252	13,946	1	,000	2,564	1,564	4,203
	Father's education(1)	-,233	,236	,976	1	,323	,792	,499	1,258
	Any combustible product (Current)(1)	2,060	,230	80,527	1	,000	7,849	5,004	12,309
	Family e-cig use(1)	1,745	,229	57,876	1	,000	5,724	3,652	8,973
	Constant	1,270	,363	12,220	1	,000	3,561		

a. Variable(s) entered on step 1: Age, Gender, Father's education, Any combustible product (Current), Family e-cig use.

NB.: For the Age variable, SPSS produced adjusted odds ratios for the inverse association (1/x), so when this was presented in the Results, the multiplicative inverse numbers are shown.

Crosstabs

Case Processing Summary

	Cases					
	Valid		Missing		Total	
	N	Percent	N	Percent	N	Percent
Age * Susceptibility to smoking?	2510	99,3%	17,426	0,7%	2527,426	100,0%
Gender * Susceptibility to smoking?	2510	99,3%	17,426	0,7%	2527,426	100,0%
Pocket money * Susceptibility to smoking?	2504[a]	99,1%	23,426	0,9%	2527,426	100,0%
Father's education * Susceptibility to smoking?	2297[a]	90,9%	230,426	9,1%	2527,426	100,0%
Mother's education * Susceptibility to smoking?	2350[a]	93,0%	177,426	7,0%	2527,426	100,0%
Ever e-cig use * Susceptibility to smoking?	2508[a]	99,2%	19,426	0,8%	2527,426	100,0%
Family e-cig use * Susceptibility to smoking?	2502[a]	99,0%	25,426	1,0%	2527,426	100,0%
Family smoking * Susceptibility to smoking?	2510[a]	99,3%	17,426	0,7%	2527,426	100,0%
Ever shisha use * Susceptibility to smoking?	2481[a]	98,2%	46,426	1,8%	2527,426	100,0%

a. Number of valid cases is different from the total count in the crosstabulation table because the cell counts have been rounded.

Age * Susceptibility to smoking?

Crosstab

			Susceptibility to smoking?		
			1	2	Total
Age	13 years old	Count	105	993	1098
		% within Age	9,6%	90,4%	100,0%

	14 years old	Count	133	732	865
		% within Age	15,4%	84,6%	100,0%
	15 years old	Count	85	462	547
		% within Age	15,5%	84,5%	100,0%
Total		Count	323	2187	2510
		% within Age	12,9%	87,1%	100,0%

Chi-Square Tests

	Value	df	Asymptotic Significance (2-sided)
Pearson Chi-Square	19,030[a]	2	,000
Likelihood Ratio	19,503	2	,000
Linear-by-Linear Association	15,159	1	,000
N of Valid Cases	2510		

a. 0 cells (,0%) have expected count less than 5. The minimum expected count is 70,39.

Risk Estimate

	Value
Odds Ratio for Age (13 years old / 14 years old)	[a]

a. Risk Estimate statistics cannot be computed. They are only computed for a 2*2 table without empty cells.

Gender * Susceptibility to smoking?

Crosstab

			Susceptibility to smoking?		
			1	2	Total
Gender	Male	Count	139	1096	1235
		% within Gender	11,3%	88,7%	100,0%

	Female	Count	184	1091	1275
		% within Gender	14,4%	85,6%	100,0%
Total		Count	323	2187	2510
		% within Gender	12,9%	87,1%	100,0%

Chi-Square Tests

	Value	df	Asymptotic Significance (2-sided)	Exact Sig. (2-sided)	Exact Sig. (1-sided)
Pearson Chi-Square	5,645[a]	1	,018		
Continuity Correction[b]	5,365	1	,021		
Likelihood Ratio	5,664	1	,017		
Fisher's Exact Test				,020	,010
Linear-by-Linear Association	5,643	1	,018		
N of Valid Cases	2510				

a. 0 cells (,0%) have expected count less than 5. The minimum expected count is 158,93.

b. Computed only for a 2x2 table

Risk Estimate

	Value	95% Confidence Interval Lower	Upper
Odds Ratio for Gender (Male / Female)	,752	,594	,952
For cohort Susceptibility to smoking? = 1	,780	,635	,958
For cohort Susceptibility to smoking? = 2	1,037	1,006	1,069
N of Valid Cases	2510		

Pocket money * Susceptibility to smoking?

Crosstab

	Susceptibility to smoking?	Total

			1	2	
Pocket money	No money	Count	12	143	155
		% within Pocket money	7,7%	92,3%	100,0%
	1-9€	Count	158	1329	1487
		% within Pocket money	10,6%	89,4%	100,0%
	10-19€	Count	121	551	672
		% within Pocket money	18,0%	82,0%	100,0%
	>20€	Count	32	158	190
		% within Pocket money	16,8%	83,2%	100,0%
Total		Count	323	2181	2504
		% within Pocket money	12,9%	87,1%	100,0%

Chi-Square Tests

	Value	df	Asymptotic Significance (2-sided)
Pearson Chi-Square	28,739[a]	3	,000
Likelihood Ratio	27,984	3	,000
Linear-by-Linear Association	23,074	1	,000
N of Valid Cases	2504		

a. 0 cells (,0%) have expected count less than 5. The minimum expected count is 19,99.

Risk Estimate

	Value
Odds Ratio for Pocket money (No money / 1-9€)	[a]

a. Risk Estimate statistics cannot be computed. They are only computed for a 2*2 table without empty cells.

Father's education * Susceptibility to smoking?

Crosstab

			Susceptibility to smoking? 1	2	Total
Father's education	High school or above	Count	230	1515	1745
		% within Father's education	13,2%	86,8%	100,0%
	Middle school or below	Count	65	487	552
		% within Father's education	11,8%	88,2%	100,0%
Total		Count	295	2002	2297
		% within Father's education	12,8%	87,2%	100,0%

Chi-Square Tests

	Value	df	Asymptotic Significance (2-sided)	Exact Sig. (2-sided)	Exact Sig. (1-sided)
Pearson Chi-Square	,740[a]	1	,390		
Continuity Correction[b]	,619	1	,431		
Likelihood Ratio	,752	1	,386		
Fisher's Exact Test				,422	,217
Linear-by-Linear Association	,739	1	,390		
N of Valid Cases	2297				

a. 0 cells (,0%) have expected count less than 5. The minimum expected count is 70,89.

b. Computed only for a 2x2 table

Risk Estimate

	Value	95% Confidence Interval Lower	Upper
Odds Ratio for Father's education (High school or above / Middle school or below)	1,137	,848	1,526
For cohort Susceptibility to smoking? = 1	1,119	,865	1,449
For cohort Susceptibility to smoking? = 2	,984	,950	1,020
N of Valid Cases	2297		

Mother's education * Susceptibility to smoking?

Crosstab

			Susceptibility to smoking?		
			1	2	Total
Mother's education	High school or above	Count	257	1642	1899
		% within Mother's education	13,5%	86,5%	100,0%
	Middle school or below	Count	52	399	451
		% within Mother's education	11,5%	88,5%	100,0%
Total		Count	309	2041	2350
		% within Mother's education	13,1%	86,9%	100,0%

Chi-Square Tests

	Value	df	Asymptotic Significance (2-sided)	Exact Sig. (2-sided)	Exact Sig. (1-sided)
Pearson Chi-Square	1,281[a]	1	,258		
Continuity Correction[b]	1,112	1	,292		
Likelihood Ratio	1,318	1	,251		
Fisher's Exact Test				,278	,146
Linear-by-Linear Association	1,280	1	,258		
N of Valid Cases	2350				

a. 0 cells (,0%) have expected count less than 5. The minimum expected count is 59,30.

b. Computed only for a 2x2 table

Risk Estimate

	Value	95% Confidence Interval	
		Lower	Upper
Odds Ratio for Mother's education (High school or above / Middle school or below)	1,201	,874	1,650

For cohort Susceptibility to smoking? = 1	1,174	,887	1,553
For cohort Susceptibility to smoking? = 2	,977	,941	1,015
N of Valid Cases	2350		

Ever e-cig use * Susceptibility to smoking?

Crosstab

			Susceptibility to smoking?		
			1	2	Total
Ever e-cig use	Yes	Count	19	42	61
		% within Ever e-cig use	31,1%	68,9%	100,0%
	No	Count	304	2143	2447
		% within Ever e-cig use	12,4%	87,6%	100,0%
Total		Count	323	2185	2508
		% within Ever e-cig use	12,9%	87,1%	100,0%

Chi-Square Tests

	Value	df	Asymptotic Significance (2-sided)	Exact Sig. (2-sided)	Exact Sig. (1-sided)
Pearson Chi-Square	18,597[a]	1	,000		
Continuity Correction[b]	16,966	1	,000		
Likelihood Ratio	14,249	1	,000		
Fisher's Exact Test				,000	,000
Linear-by-Linear Association	18,590	1	,000		
N of Valid Cases	2508				

a. 0 cells (,0%) have expected count less than 5. The minimum expected count is 7,86.

b. Computed only for a 2x2 table

Risk Estimate

	Value	95% Confidence Interval	
		Lower	Upper
Odds Ratio for Ever e-cig use (Yes / No)	3,189	1,831	5,555
For cohort Susceptibility to smoking? = 1	2,507	1,701	3,694
For cohort Susceptibility to smoking? = 2	,786	,664	,931
N of Valid Cases	2508		

Family e-cig use * Susceptibility to smoking?

Crosstab

			Susceptibility to smoking?		Total
			1	2	
Family e-cig use	Yes	Count	36	209	245
		% within Family e-cig use	14,7%	85,3%	100,0%
	No	Count	286	1971	2257
		% within Family e-cig use	12,7%	87,3%	100,0%
Total		Count	322	2180	2502
		% within Family e-cig use	12,9%	87,1%	100,0%

Chi-Square Tests

	Value	df	Asymptotic Significance (2-sided)	Exact Sig. (2-sided)	Exact Sig. (1-sided)
Pearson Chi-Square	,806[a]	1	,369		
Continuity Correction[b]	,636	1	,425		
Likelihood Ratio	,779	1	,377		
Fisher's Exact Test				,366	,210
Linear-by-Linear Association	,806	1	,369		
N of Valid Cases	2502				

a. 0 cells (,0%) have expected count less than 5. The minimum expected count is 31,53.

b. Computed only for a 2x2 table

Risk Estimate

	Value	95% Confidence Interval Lower	Upper
Odds Ratio for Family e-cig use (Yes / No)	1,187	,816	1,727
For cohort Susceptibility to smoking? = 1	1,160	,842	1,598
For cohort Susceptibility to smoking? = 2	,977	,925	1,031
N of Valid Cases	2502		

Family smoking * Susceptibility to smoking?

Crosstab

			Susceptibility to smoking? 1	2	Total
Family smoking	Someone	Count	322	2183	2505
		% within Family smoking	12,9%	87,1%	100,0%
	No one	Count	1	4	5
		% within Family smoking	20,0%	80,0%	100,0%
Total		Count	323	2187	2510
		% within Family smoking	12,9%	87,1%	100,0%

Chi-Square Tests

	Value	df	Asymptotic Significance (2-sided)	Exact Sig. (2-sided)	Exact Sig. (1-sided)
Pearson Chi-Square	,227[a]	1	,634		
Continuity Correction[b]	,000	1	1,000		
Likelihood Ratio	,199	1	,655		
Fisher's Exact Test				,498	,498

Linear-by-Linear Association	,227	1	,634		
N of Valid Cases	2510				

a. 2 cells (50,0%) have expected count less than 5. The minimum expected count is ,64.

b. Computed only for a 2x2 table

Risk Estimate

	Value	95% Confidence Interval	
		Lower	Upper
Odds Ratio for Family smoking (Someone / No one)	,590	,066	5,295
For cohort Susceptibility to smoking? = 1	,643	,111	3,721
For cohort Susceptibility to smoking? = 2	1,089	,703	1,689
N of Valid Cases	2510		

Logistic Regression

Case Processing Summary

Unweighted Cases[a]		N	Percent
Selected Cases	Included in Analysis	2491	98,6
	Missing Cases	35	1,4
	Total	2526	100,0
Unselected Cases		0	,0
Total		2526	100,0

a. If weight is in effect, see classification table for the total number of cases.

Dependent Variable Encoding

Original Value	Internal Value
1	0
2	1

Categorical Variables Codings

		Frequency	Parameter coding (1)	(2)	(3)
Pocket money	No money	167	,000	,000	,000
	1-9€	1487	1,000	,000	,000
	10-19€	656	,000	1,000	,000
	>20€	181	,000	,000	1,000
Age	13 years old	1091	,000	,000	
	14 years old	844	1,000	,000	
	15 years old	556	,000	1,000	
Ever e-cig use	Yes	60	,000		
	No	2431	1,000		
Gender	Male	1204	,000		
	Female	1287	1,000		

Block 0: Beginning Block

Classification Table[a,b]

	Observed		Predicted: Susceptibility to smoking? 1	2	Percentage Correct
Step 0	Susceptibility to smoking?	1	0	321	,0
		2	0	2178	100,0
	Overall Percentage				87,2

a. Constant is included in the model.

b. The cut value is ,500

Variables in the Equation

		B	S.E.	Wald	df	Sig.	Exp(B)
Step 0	Constant	1,915	,060	1025,831	1	,000	6,784

Variables not in the Equation

			Score	df	Sig.
Step 0	Variables	Age	19,155	2	,000
		Age(1)	7,371	1	,007
		Age(2)	4,546	1	,033
		Gender(1)	5,571	1	,018
		Pocket money	29,123	3	,000
		Pocket money(1)	17,047	1	,000
		Pocket money(2)	21,675	1	,000
		Pocket money(3)	2,839	1	,092
		Ever e-cig use(1)	18,317	1	,000
	Overall Statistics		65,849	7	,000

Block 1: Method = Enter

Omnibus Tests of Model Coefficients

		Chi-square	df	Sig.
Step 1	Step	62,050	7	,000
	Block	62,050	7	,000
	Model	62,050	7	,000

Model Summary

Step	-2 Log likelihood	Cox & Snell R Square	Nagelkerke R Square
1	1854,831[a]	,025	,046

a. Estimation terminated at iteration number 5 because parameter estimates changed by less than ,001.

Hosmer and Lemeshow Test

Step	Chi-square	df	Sig.
1	6,968	7	,432

Contingency Table for Hosmer and Lemeshow Test

		Susceptibility to smoking? = 1		Susceptibility to smoking? = 2		
		Observed	Expected	Observed	Expected	Total
Step 1	1	66	61,869	187	191,466	253
	2	48	47,019	216	217,076	264
	3	32	35,650	203	200,074	236
	4	22	22,769	140	139,270	162
	5	29	35,400	226	218,913	254
	6	31	30,317	248	248,972	279
	7	32	26,174	218	223,804	250
	8	38	31,348	306	311,853	343
	9	24	30,547	434	426,956	458

Classification Table[a]

	Observed		Predicted		
			Susceptibility to smoking?		Percentage
			1	2	Correct
Step 1	Susceptibility to smoking?	1	0	321	,0
		2	0	2178	100,0
	Overall Percentage				87,2

a. The cut value is ,500

Variables in the Equation

		B	S.E.	Wald	df	Sig.	Exp(B)	95% C.I.for EXP(B)	
								Lower	Upper
Step 1[a]	Age			13,937	2	,001			
	Age(1)	-,475	,142	11,255	1	,001	,622	,471	,821
	Age(2)	-,486	,159	9,365	1	,002	,615	,450	,840
	Gender(1)	-,324	,123	6,983	1	,008	,723	,568	,920
	Pocket money			22,613	3	,000			
	Pocket money(1)	-,321	,317	1,025	1	,311	,726	,390	1,350
	Pocket money(2)	-,881	,322	7,474	1	,006	,415	,221	,779
	Pocket money(3)	-,808	,364	4,937	1	,026	,446	,219	,909
	Ever e-cig use(1)	1,117	,292	14,675	1	,000	3,056	1,726	5,412
	Constant	1,825	,415	19,367	1	,000	6,206		

a. Variable(s) entered on step 1: Age, Gender, Pocket money, Ever e-cig use.

NB.: For the Age and Pocket money variables, SPSS produced adjusted odds ratios for the inverse association (1/x), so when these were presented in the Results, the multiplicative inverse numbers are shown.
