# Supplementary material for: Prevalence of Electronic Cigarette Use and Its Determinants among 13-to-15-Year-Old Students in Greece: Results from the 2013 Global Youth Tobacco Survey (GYTS)
Source: Int J Environ Res Public Health. 2020 Mar 4;17(5):1671. doi: 10.3390/ijerph17051671 (PMC7084902; doi:10.3390/ijerph17051671)
Supplement: Supplementary file 1 [file ijerph-17-01671-s001.zip › Output of statistical analysis.docx]

**Frequencies**

| **Statistics** | | | | | | | | | | | | | | |
| --- | --- | --- | --- | --- | --- | --- | --- | --- | --- | --- | --- | --- | --- | --- |
|  | | Age | Gender | Pocket money | Father's education | Mother's education | Ever tobacco use | Tobacco smoking | Ever e-cig use | E-cigarette use | Family e-cig use | Family smoking | Smoking intention | Ever shisha use |
| N | Valid | 4096 | 4095 | 4084 | 3749 | 3837 | 4034 | 3991 | 4066 | 4057 | 4058 | 4096 | 4057 | 4030 |
|  | Missing | 0 | 1 | 12 | 347 | 259 | 62 | 105 | 30 | 39 | 38 | 0 | 39 | 66 |

**Frequency Table**

| **Age** | | | | | |
| --- | --- | --- | --- | --- | --- |
|  | | Frequency | Percent | Valid Percent | Cumulative Percent |
| Valid | 13 years old | 1542 | 37,7 | 37,7 | 37,7 |
|  | 14 years old | 1432 | 35,0 | 35,0 | 72,6 |
|  | 15 years old | 1122 | 27,4 | 27,4 | 100,0 |
|  | Total | 4096 | 100,0 | 100,0 |  |

| **Gender** | | | | | |
| --- | --- | --- | --- | --- | --- |
|  | | Frequency | Percent | Valid Percent | Cumulative Percent |
| Valid | Male | 2108 | 51,5 | 51,5 | 51,5 |
|  | Female | 1987 | 48,5 | 48,5 | 100,0 |
|  | Total | 4095 | 100,0 | 100,0 |  |
| Missing | System | 1 | ,0 |  |  |
| Total | | 4096 | 100,0 |  |  |

| **Pocket money** | | | | | |
| --- | --- | --- | --- | --- | --- |
|  | | Frequency | Percent | Valid Percent | Cumulative Percent |
| Valid | No money | 241 | 5,9 | 5,9 | 5,9 |
|  | 1-9€ | 2245 | 54,8 | 55,0 | 60,9 |
|  | 10-19€ | 1162 | 28,4 | 28,5 | 89,3 |
|  | >20€ | 436 | 10,7 | 10,7 | 100,0 |
|  | Total | 4084 | 99,7 | 100,0 |  |
| Missing | System | 12 | ,3 |  |  |
| Total | | 4096 | 100,0 |  |  |

| **Father's education** | | | | | |
| --- | --- | --- | --- | --- | --- |
|  | | Frequency | Percent | Valid Percent | Cumulative Percent |
| Valid | High school or above | 2707 | 66,1 | 72,2 | 72,2 |
|  | Middle school or below | 1042 | 25,5 | 27,8 | 100,0 |
|  | Total | 3749 | 91,5 | 100,0 |  |
| Missing | System | 347 | 8,5 |  |  |
| Total | | 4096 | 100,0 |  |  |

| **Mother's education** | | | | | |
| --- | --- | --- | --- | --- | --- |
|  | | Frequency | Percent | Valid Percent | Cumulative Percent |
| Valid | High school or above | 2997 | 73,2 | 78,1 | 78,1 |
|  | Middle school or below | 840 | 20,5 | 21,9 | 100,0 |
|  | Total | 3837 | 93,7 | 100,0 |  |
| Missing | System | 259 | 6,3 |  |  |
| Total | | 4096 | 100,0 |  |  |

| **Ever tobacco use** | | | | | |
| --- | --- | --- | --- | --- | --- |
|  | | Frequency | Percent | Valid Percent | Cumulative Percent |
| Valid | Yes | 1228 | 30,0 | 30,4 | 30,4 |
|  | No | 2806 | 68,5 | 69,6 | 100,0 |
|  | Total | 4034 | 98,5 | 100,0 |  |
| Missing | System | 62 | 1,5 |  |  |
| Total | | 4096 | 100,0 |  |  |

| **Tobacco smoking** | | | | | |
| --- | --- | --- | --- | --- | --- |
|  | | Frequency | Percent | Valid Percent | Cumulative Percent |
| Valid | Yes | 402 | 9,8 | 10,1 | 10,1 |
|  | No | 3588 | 87,6 | 89,9 | 100,0 |
|  | Total | 3991 | 97,4 | 100,0 |  |
| Missing | System | 105 | 2,6 |  |  |
| Total | | 4096 | 100,0 |  |  |

| **Ever e-cig use** | | | | | |
| --- | --- | --- | --- | --- | --- |
|  | | Frequency | Percent | Valid Percent | Cumulative Percent |
| Valid | Yes | 498 | 12,2 | 12,3 | 12,3 |
|  | No | 3568 | 87,1 | 87,7 | 100,0 |
|  | Total | 4066 | 99,3 | 100,0 |  |
| Missing | System | 30 | ,7 |  |  |
| Total | | 4096 | 100,0 |  |  |

| **E-cigarette use** | | | | | |
| --- | --- | --- | --- | --- | --- |
|  | | Frequency | Percent | Valid Percent | Cumulative Percent |
| Valid | Yes | 115 | 2,8 | 2,8 | 2,8 |
|  | No | 3942 | 96,2 | 97,2 | 100,0 |
|  | Total | 4057 | 99,0 | 100,0 |  |
| Missing | System | 39 | 1,0 |  |  |
| Total | | 4096 | 100,0 |  |  |

| **Family e-cig use** | | | | | |
| --- | --- | --- | --- | --- | --- |
|  | | Frequency | Percent | Valid Percent | Cumulative Percent |
| Valid | Yes | 498 | 12,2 | 12,3 | 12,3 |
|  | No | 3560 | 86,9 | 87,7 | 100,0 |
|  | Total | 4058 | 99,1 | 100,0 |  |
| Missing | System | 38 | ,9 |  |  |
| Total | | 4096 | 100,0 |  |  |

| **Family smoking** | | | | | |
| --- | --- | --- | --- | --- | --- |
|  | | Frequency | Percent | Valid Percent | Cumulative Percent |
| Valid | Someone | 4077 | 99,5 | 99,5 | 99,5 |
|  | No one | 19 | ,5 | ,5 | 100,0 |
|  | Total | 4096 | 100,0 | 100,0 |  |

**Frequency Table**

| **Susceptibility to smoking?** | | | | | |
| --- | --- | --- | --- | --- | --- |
|  | | Frequency | Percent | Valid Percent | Cumulative Percent |
| Valid | 1 | 323 | 12,8 | 12,9 | 12,9 |
|  | 2 | 2187 | 86,5 | 87,1 | 100,0 |
|  | Total | 2510 | 99,3 | 100,0 |  |
| Missing | System | 17 | ,7 |  |  |
| Total | | 2527 | 100,0 |  |  |

**T-Test**

| **One-Sample Statistics** | | | | |
| --- | --- | --- | --- | --- |
|  | N | Mean | Std. Deviation | Std. Error Mean |
| Gender | 4095 | 1,49 | ,500 | ,008 |
| Father's education | 3749 | 1,28 | ,448 | ,007 |
| Mother's education | 3837 | 1,22 | ,414 | ,007 |
| Ever tobacco use | 4034 | 1,70 | ,460 | ,007 |
| Tobacco smoking | 3991 | 1,90 | ,301 | ,005 |
| Ever e-cig use | 4066 | 1,88 | ,328 | ,005 |
| E-cigarette use | 4057 | 1,97 | ,166 | ,003 |
| Family e-cig use | 4058 | 1,88 | ,328 | ,005 |
| Family smoking | 4096 | 1,00 | ,068 | ,001 |
| Susceptibility to smoking? | 4058 | 1,70 | ,460 | ,007 |
| Ever shisha use | 4030 | 1,84 | ,370 | ,006 |

| **One-Sample Test** | | | | | | |
| --- | --- | --- | --- | --- | --- | --- |
|  | Test Value = 1 | | | | | |
|  | t | df | Sig. (2-tailed) | Mean Difference | 95% Confidence Interval of the Difference | |
|  |  |  |  |  | Lower | Upper |
| Gender | 62,111 | 4094 | ,000 | ,485 | ,47 | ,50 |
| Father's education | 37,994 | 3748 | ,000 | ,278 | ,26 | ,29 |
| Mother's education | 32,781 | 3836 | ,000 | ,219 | ,21 | ,23 |
| Ever tobacco use | 95,999 | 4033 | ,000 | ,696 | ,68 | ,71 |
| Tobacco smoking | 188,611 | 3990 | ,000 | ,899 | ,89 | ,91 |
| Ever e-cig use | 170,629 | 4065 | ,000 | ,877 | ,87 | ,89 |
| E-cigarette use | 373,170 | 4056 | ,000 | ,972 | ,97 | ,98 |
| Family e-cig use | 170,267 | 4057 | ,000 | ,877 | ,87 | ,89 |
| Family smoking | 4,389 | 4095 | ,000 | ,005 | ,00 | ,01 |
| Susceptibility to smoking? | 96,321 | 4057 | ,000 | ,696 | ,68 | ,71 |
| Ever shisha use | 143,417 | 4029 | ,000 | ,836 | ,82 | ,85 |

**T-Test**

| **One-Sample Statistics** | | | | |
| --- | --- | --- | --- | --- |
|  | N | Mean | Std. Deviation | Std. Error Mean |
| Susceptibility to smoking? | 2510 | 1,87 | ,335 | ,007 |

| **One-Sample Test** | | | | | | |
| --- | --- | --- | --- | --- | --- | --- |
|  | Test Value = 1 | | | | | |
|  | t | df | Sig. (2-tailed) | Mean Difference | 95% Confidence Interval of the Difference | |
|  |  |  |  |  | Lower | Upper |
| Susceptibility to smoking? | 130,325 | 2509 | ,000 | ,871 | ,86 | ,88 |

**Frequencies**

| **Statistics** | | | | | | | | | | | | | |
| --- | --- | --- | --- | --- | --- | --- | --- | --- | --- | --- | --- | --- | --- |
|  | | Ever tobacco use | Tobacco smoking | Ever other smoked tobacco product | Other smoked tobacco product | Ever other non-smoke tobacco | Non-smoked tobacco product | Ever e-cig use | E-cigarette use | Any combustible product (Ever) | Any nicotine-containing product (Ever) | Any combustible product (Current) | Any nicotine-containing product (Current) |
| N | Valid | 4034 | 3991 | 3960 | 3951 | 4045 | 4048 | 4066 | 4057 | 3974 | 3976 | 3900 | 3883 |
|  | Missing | 62 | 105 | 136 | 145 | 51 | 48 | 30 | 39 | 122 | 120 | 196 | 213 |

**Frequency Table**

| **Ever tobacco use** | | | | | |
| --- | --- | --- | --- | --- | --- |
|  | | Frequency | Percent | Valid Percent | Cumulative Percent |
| Valid | Yes | 1228 | 30,0 | 30,4 | 30,4 |
|  | No | 2806 | 68,5 | 69,6 | 100,0 |
|  | Total | 4034 | 98,5 | 100,0 |  |
| Missing | System | 62 | 1,5 |  |  |
| Total | | 4096 | 100,0 |  |  |

| **Tobacco smoking** | | | | | |
| --- | --- | --- | --- | --- | --- |
|  | | Frequency | Percent | Valid Percent | Cumulative Percent |
| Valid | Yes | 402 | 9,8 | 10,1 | 10,1 |
|  | No | 3588 | 87,6 | 89,9 | 100,0 |
|  | Total | 3991 | 97,4 | 100,0 |  |
| Missing | System | 105 | 2,6 |  |  |
| Total | | 4096 | 100,0 |  |  |

| **Ever other smoked tobacco product** | | | | | |
| --- | --- | --- | --- | --- | --- |
|  | | Frequency | Percent | Valid Percent | Cumulative Percent |
| Valid | Yes | 663 | 16,2 | 16,7 | 16,7 |
|  | No | 3296 | 80,5 | 83,3 | 100,0 |
|  | Total | 3960 | 96,7 | 100,0 |  |
| Missing | System | 136 | 3,3 |  |  |
| Total | | 4096 | 100,0 |  |  |

| **Other smoked tobacco product** | | | | | |
| --- | --- | --- | --- | --- | --- |
|  | | Frequency | Percent | Valid Percent | Cumulative Percent |
| Valid | Yes | 301 | 7,3 | 7,6 | 7,6 |
|  | No | 3650 | 89,1 | 92,4 | 100,0 |
|  | Total | 3951 | 96,5 | 100,0 |  |
| Missing | System | 145 | 3,5 |  |  |
| Total | | 4096 | 100,0 |  |  |

| **Ever other non-smoke tobacco** | | | | | |
| --- | --- | --- | --- | --- | --- |
|  | | Frequency | Percent | Valid Percent | Cumulative Percent |
| Valid | Yes | 137 | 3,3 | 3,4 | 3,4 |
|  | No | 3908 | 95,4 | 96,6 | 100,0 |
|  | Total | 4045 | 98,8 | 100,0 |  |
| Missing | System | 51 | 1,2 |  |  |
| Total | | 4096 | 100,0 |  |  |

| **Non-smoked tobacco product** | | | | | |
| --- | --- | --- | --- | --- | --- |
|  | | Frequency | Percent | Valid Percent | Cumulative Percent |
| Valid | Yes | 61 | 1,5 | 1,5 | 1,5 |
|  | No | 3987 | 97,3 | 98,5 | 100,0 |
|  | Total | 4048 | 98,8 | 100,0 |  |
| Missing | System | 48 | 1,2 |  |  |
| Total | | 4096 | 100,0 |  |  |

| **Ever e-cig use** | | | | | |
| --- | --- | --- | --- | --- | --- |
|  | | Frequency | Percent | Valid Percent | Cumulative Percent |
| Valid | Yes | 498 | 12,2 | 12,3 | 12,3 |
|  | No | 3568 | 87,1 | 87,7 | 100,0 |
|  | Total | 4066 | 99,3 | 100,0 |  |
| Missing | System | 30 | ,7 |  |  |
| Total | | 4096 | 100,0 |  |  |

| **E-cigarette use** | | | | | |
| --- | --- | --- | --- | --- | --- |
|  | | Frequency | Percent | Valid Percent | Cumulative Percent |
| Valid | Yes | 115 | 2,8 | 2,8 | 2,8 |
|  | No | 3942 | 96,2 | 97,2 | 100,0 |
|  | Total | 4057 | 99,0 | 100,0 |  |
| Missing | System | 39 | 1,0 |  |  |
| Total | | 4096 | 100,0 |  |  |

| **Any combustible product (Ever)** | | | | | |
| --- | --- | --- | --- | --- | --- |
|  | | Frequency | Percent | Valid Percent | Cumulative Percent |
| Valid | 1 | 1386 | 33,8 | 34,9 | 34,9 |
|  | 2 | 2588 | 63,2 | 65,1 | 100,0 |
|  | Total | 3974 | 97,0 | 100,0 |  |
| Missing | System | 122 | 3,0 |  |  |
| Total | | 4096 | 100,0 |  |  |

| **Any nicotine-containing product (Ever)** | | | | | |
| --- | --- | --- | --- | --- | --- |
|  | | Frequency | Percent | Valid Percent | Cumulative Percent |
| Valid | 1 | 1511 | 36,9 | 38,0 | 38,0 |
|  | 2 | 2464 | 60,2 | 62,0 | 100,0 |
|  | Total | 3976 | 97,1 | 100,0 |  |
| Missing | System | 120 | 2,9 |  |  |
| Total | | 4096 | 100,0 |  |  |

| **Any combustible product (Current)** | | | | | |
| --- | --- | --- | --- | --- | --- |
|  | | Frequency | Percent | Valid Percent | Cumulative Percent |
| Valid | Yes | 518 | 12,6 | 13,3 | 13,3 |
|  | No | 3382 | 82,6 | 86,7 | 100,0 |
|  | Total | 3900 | 95,2 | 100,0 |  |
| Missing | System | 196 | 4,8 |  |  |
| Total | | 4096 | 100,0 |  |  |

| **Any nicotine-containing product (Current)** | | | | | |
| --- | --- | --- | --- | --- | --- |
|  | | Frequency | Percent | Valid Percent | Cumulative Percent |
| Valid | Yes | 592 | 14,4 | 15,2 | 15,2 |
|  | No | 3291 | 80,3 | 84,8 | 100,0 |
|  | Total | 3883 | 94,8 | 100,0 |  |
| Missing | System | 213 | 5,2 |  |  |
| Total | | 4096 | 100,0 |  |  |

**Crosstabs**

| **Case Processing Summary** | | | | | | |
| --- | --- | --- | --- | --- | --- | --- |
|  | Cases | | | | | |
|  | Valid | | Missing | | Total | |
|  | N | Percent | N | Percent | N | Percent |
| Age * Tobacco smoking | 3990 | 97,4% | 106,000 | 2,6% | 4096 | 100,0% |
| Age * Other smoked tobacco product | 3952^a^ | 96,5% | 144,000 | 3,5% | 4096 | 100,0% |
| Age * Non-smoked tobacco product | 4048^a^ | 98,8% | 48,000 | 1,2% | 4096 | 100,0% |
| Age * E-cigarette use | 4057^a^ | 99,0% | 39,000 | 1,0% | 4096 | 100,0% |
| Age * Any combustible product (Current) | 3901^a^ | 95,2% | 195,000 | 4,8% | 4096 | 100,0% |
| Age * Any nicotine-containing product (Current) | 3882^a^ | 94,8% | 214,000 | 5,2% | 4096 | 100,0% |
| a. Number of valid cases is different from the total count in the crosstabulation table because the cell counts have been rounded. | | | | | | |

| **Age * Tobacco smoking Crosstabulation** | | | | | |
| --- | --- | --- | --- | --- | --- |
|  | | | Tobacco smoking | | Total |
|  |  |  | Yes | No |  |
| Age | 13 years old | Count | 71 | 1437 | 1508 |
|  |  | % within Age | 4,7% | 95,3% | 100,0% |
|  | 14 years old | Count | 147 | 1247 | 1394 |
|  |  | % within Age | 10,5% | 89,5% | 100,0% |
|  | 15 years old | Count | 184 | 904 | 1088 |
|  |  | % within Age | 16,9% | 83,1% | 100,0% |
| Total | | Count | 402 | 3588 | 3990 |
|  |  | % within Age | 10,1% | 89,9% | 100,0% |

| **Age * Other smoked tobacco product Crosstabulation** | | | | | |
| --- | --- | --- | --- | --- | --- |
|  | | | Other smoked tobacco product | | Total |
|  |  |  | Yes | No |  |
| Age | 13 years old | Count | 41 | 1443 | 1484 |
|  |  | % within Age | 2,8% | 97,2% | 100,0% |
|  | 14 years old | Count | 119 | 1263 | 1382 |
|  |  | % within Age | 8,6% | 91,4% | 100,0% |
|  | 15 years old | Count | 141 | 945 | 1086 |
|  |  | % within Age | 13,0% | 87,0% | 100,0% |
| Total | | Count | 301 | 3651 | 3952 |
|  |  | % within Age | 7,6% | 92,4% | 100,0% |

| **Age * Non-smoked tobacco product Crosstabulation** | | | | | |
| --- | --- | --- | --- | --- | --- |
|  | | | Non-smoked tobacco product | | Total |
|  |  |  | Yes | No |  |
| Age | 13 years old | Count | 15 | 1502 | 1517 |
|  |  | % within Age | 1,0% | 99,0% | 100,0% |
|  | 14 years old | Count | 23 | 1395 | 1418 |
|  |  | % within Age | 1,6% | 98,4% | 100,0% |
|  | 15 years old | Count | 23 | 1090 | 1113 |
|  |  | % within Age | 2,1% | 97,9% | 100,0% |
| Total | | Count | 61 | 3987 | 4048 |
|  |  | % within Age | 1,5% | 98,5% | 100,0% |

| **Age * E-cigarette use Crosstabulation** | | | | | |
| --- | --- | --- | --- | --- | --- |
|  | | | E-cigarette use | | Total |
|  |  |  | Yes | No |  |
| Age | 13 years old | Count | 17 | 1502 | 1519 |
|  |  | % within Age | 1,1% | 98,9% | 100,0% |
|  | 14 years old | Count | 46 | 1377 | 1423 |
|  |  | % within Age | 3,2% | 96,8% | 100,0% |
|  | 15 years old | Count | 52 | 1063 | 1115 |
|  |  | % within Age | 4,7% | 95,3% | 100,0% |
| Total | | Count | 115 | 3942 | 4057 |
|  |  | % within Age | 2,8% | 97,2% | 100,0% |

| **Age * Any combustible product (Current) Crosstabulation** | | | | | |
| --- | --- | --- | --- | --- | --- |
|  | | | Any combustible product (Current) | | Total |
|  |  |  | Yes | No |  |
| Age | 13 years old | Count | 83 | 1379 | 1462 |
|  |  | % within Age | 5,7% | 94,3% | 100,0% |
|  | 14 years old | Count | 193 | 1167 | 1360 |
|  |  | % within Age | 14,2% | 85,8% | 100,0% |
|  | 15 years old | Count | 242 | 837 | 1079 |
|  |  | % within Age | 22,4% | 77,6% | 100,0% |
| Total | | Count | 518 | 3383 | 3901 |
|  |  | % within Age | 13,3% | 86,7% | 100,0% |

| **Age * Any nicotine-containing product (Current) Crosstabulation** | | | | | |
| --- | --- | --- | --- | --- | --- |
|  | | | Any nicotine-containing product (Current) | | Total |
|  |  |  | Yes | No |  |
| Age | 13 years old | Count | 105 | 1335 | 1440 |
|  |  | % within Age | 7,3% | 92,7% | 100,0% |
|  | 14 years old | Count | 223 | 1143 | 1366 |
|  |  | % within Age | 16,3% | 83,7% | 100,0% |
|  | 15 years old | Count | 263 | 813 | 1076 |
|  |  | % within Age | 24,4% | 75,6% | 100,0% |
| Total | | Count | 591 | 3291 | 3882 |
|  |  | % within Age | 15,2% | 84,8% | 100,0% |

**Crosstabs**

| **Case Processing Summary** | | | | | | |
| --- | --- | --- | --- | --- | --- | --- |
|  | Cases | | | | | |
|  | Valid | | Missing | | Total | |
|  | N | Percent | N | Percent | N | Percent |
| Age * E-cigarette use | 4057 | 99,0% | 39,000 | 1,0% | 4096 | 100,0% |
| Gender * E-cigarette use | 4056^a^ | 99,0% | 40,000 | 1,0% | 4096 | 100,0% |
| Pocket money * E-cigarette use | 4047^a^ | 98,8% | 49,000 | 1,2% | 4096 | 100,0% |
| Father's education * E-cigarette use | 3715^a^ | 90,7% | 381,000 | 9,3% | 4096 | 100,0% |
| Mother's education * E-cigarette use | 3803^a^ | 92,8% | 293,000 | 7,2% | 4096 | 100,0% |
| Family e-cig use * E-cigarette use | 4032^a^ | 98,4% | 64,000 | 1,6% | 4096 | 100,0% |
| Family smoking * E-cigarette use | 4055^a^ | 99,0% | 41,000 | 1,0% | 4096 | 100,0% |
| Ever shisha use * E-cigarette use | 3995^a^ | 97,5% | 101,000 | 2,5% | 4096 | 100,0% |
| Tobacco smoking * E-cigarette use | 3958^a^ | 96,6% | 138,000 | 3,4% | 4096 | 100,0% |
| a. Number of valid cases is different from the total count in the crosstabulation table because the cell counts have been rounded. | | | | | | |

| **Crosstab** | | | | | |
| --- | --- | --- | --- | --- | --- |
|  | | | E-cigarette use | | Total |
|  |  |  | Yes | No |  |
| Age | 13 years old | Count | 17 | 1502 | 1519 |
|  |  | % within Age | 1,1% | 98,9% | 100,0% |
|  | 14 years old | Count | 46 | 1377 | 1423 |
|  |  | % within Age | 3,2% | 96,8% | 100,0% |
|  | 15 years old | Count | 52 | 1063 | 1115 |
|  |  | % within Age | 4,7% | 95,3% | 100,0% |
| Total | | Count | 115 | 3942 | 4057 |
|  |  | % within Age | 2,8% | 97,2% | 100,0% |

| **Chi-Square Tests** | | | |
| --- | --- | --- | --- |
|  | Value | df | Asymptotic Significance (2-sided) |
| Pearson Chi-Square | 30,592^a^ | 2 | ,000 |
| Likelihood Ratio | 33,139 | 2 | ,000 |
| Linear-by-Linear Association | 30,197 | 1 | ,000 |
| N of Valid Cases | 4057 |  |  |
| a. 0 cells (,0%) have expected count less than 5. The minimum expected count is 31,61. | | | |

| **Risk Estimate** | |
| --- | --- |
|  | Value |
| Odds Ratio for Age (13 years old / 14 years old) | ^a^ |
| a. Risk Estimate statistics cannot be computed. They are only computed for a 2*2 table without empty cells. | |

**Gender * E-cigarette use**

| **Crosstab** | | | | | |
| --- | --- | --- | --- | --- | --- |
|  | | | E-cigarette use | | Total |
|  |  |  | Yes | No |  |
| Gender | Male | Count | 82 | 2004 | 2086 |
|  |  | % within Gender | 3,9% | 96,1% | 100,0% |
|  | Female | Count | 33 | 1937 | 1970 |
|  |  | % within Gender | 1,7% | 98,3% | 100,0% |
| Total | | Count | 115 | 3941 | 4056 |
|  |  | % within Gender | 2,8% | 97,2% | 100,0% |

| **Chi-Square Tests** | | | | | |
| --- | --- | --- | --- | --- | --- |
|  | Value | df | Asymptotic Significance (2-sided) | Exact Sig. (2-sided) | Exact Sig. (1-sided) |
| Pearson Chi-Square | 18,715^a^ | 1 | ,000 |  |  |
| Continuity Correction^b^ | 17,905 | 1 | ,000 |  |  |
| Likelihood Ratio | 19,382 | 1 | ,000 |  |  |
| Fisher's Exact Test |  |  |  | ,000 | ,000 |
| Linear-by-Linear Association | 18,710 | 1 | ,000 |  |  |
| N of Valid Cases | 4056 |  |  |  |  |
| a. 0 cells (,0%) have expected count less than 5. The minimum expected count is 55,86. | | | | | |
| b. Computed only for a 2x2 table | | | | | |

| **Risk Estimate** | | | |
| --- | --- | --- | --- |
|  | Value | 95% Confidence Interval | |
|  |  | Lower | Upper |
| Odds Ratio for Gender (Male / Female) | 2,402 | 1,596 | 3,615 |
| For cohort E-cigarette use = Yes | 2,347 | 1,574 | 3,498 |
| For cohort E-cigarette use = No | ,977 | ,967 | ,987 |
| N of Valid Cases | 4056 |  |  |

**Pocket money * E-cigarette use**

| **Crosstab** | | | | | |
| --- | --- | --- | --- | --- | --- |
|  | | | E-cigarette use | | Total |
|  |  |  | Yes | No |  |
| Pocket money | No money | Count | 5 | 231 | 236 |
|  |  | % within Pocket money | 2,1% | 97,9% | 100,0% |
|  | 1-9€ | Count | 52 | 2170 | 2222 |
|  |  | % within Pocket money | 2,3% | 97,7% | 100,0% |
|  | 10-19€ | Count | 38 | 1117 | 1155 |
|  |  | % within Pocket money | 3,3% | 96,7% | 100,0% |
|  | >20€ | Count | 17 | 417 | 434 |
|  |  | % within Pocket money | 3,9% | 96,1% | 100,0% |
| Total | | Count | 112 | 3935 | 4047 |
|  |  | % within Pocket money | 2,8% | 97,2% | 100,0% |

| **Chi-Square Tests** | | | |
| --- | --- | --- | --- |
|  | Value | df | Asymptotic Significance (2-sided) |
| Pearson Chi-Square | 5,180^a^ | 3 | ,159 |
| Likelihood Ratio | 4,989 | 3 | ,173 |
| Linear-by-Linear Association | 4,892 | 1 | ,027 |
| N of Valid Cases | 4047 |  |  |
| a. 0 cells (,0%) have expected count less than 5. The minimum expected count is 6,53. | | | |

| **Risk Estimate** | |
| --- | --- |
|  | Value |
| Odds Ratio for Pocket money (No money / 1-9€) | ^a^ |
| a. Risk Estimate statistics cannot be computed. They are only computed for a 2*2 table without empty cells. | |

**Father's education * E-cigarette use**

| **Crosstab** | | | | | |
| --- | --- | --- | --- | --- | --- |
|  | | | E-cigarette use | | Total |
|  |  |  | Yes | No |  |
| Father's education | High school or above | Count | 66 | 2618 | 2684 |
|  |  | % within Father's education | 2,5% | 97,5% | 100,0% |
|  | Middle school or below | Count | 39 | 992 | 1031 |
|  |  | % within Father's education | 3,8% | 96,2% | 100,0% |
| Total | | Count | 105 | 3610 | 3715 |
|  |  | % within Father's education | 2,8% | 97,2% | 100,0% |

| **Chi-Square Tests** | | | | | |
| --- | --- | --- | --- | --- | --- |
|  | Value | df | Asymptotic Significance (2-sided) | Exact Sig. (2-sided) | Exact Sig. (1-sided) |
| Pearson Chi-Square | 4,752^a^ | 1 | ,029 |  |  |
| Continuity Correction^b^ | 4,282 | 1 | ,039 |  |  |
| Likelihood Ratio | 4,489 | 1 | ,034 |  |  |
| Fisher's Exact Test |  |  |  | ,035 | ,021 |
| Linear-by-Linear Association | 4,751 | 1 | ,029 |  |  |
| N of Valid Cases | 3715 |  |  |  |  |
| a. 0 cells (,0%) have expected count less than 5. The minimum expected count is 29,14. | | | | | |
| b. Computed only for a 2x2 table | | | | | |

| **Risk Estimate** | | | |
| --- | --- | --- | --- |
|  | Value | 95% Confidence Interval | |
|  |  | Lower | Upper |
| Odds Ratio for Father's education (High school or above / Middle school or below) | ,641 | ,429 | ,959 |
| For cohort E-cigarette use = Yes | ,650 | ,440 | ,959 |
| For cohort E-cigarette use = No | 1,014 | 1,000 | 1,028 |
| N of Valid Cases | 3715 |  |  |

**Mother's education * E-cigarette use**

| **Crosstab** | | | | | |
| --- | --- | --- | --- | --- | --- |
|  | | | E-cigarette use | | Total |
|  |  |  | Yes | No |  |
| Mother's education | High school or above | Count | 80 | 2894 | 2974 |
|  |  | % within Mother's education | 2,7% | 97,3% | 100,0% |
|  | Middle school or below | Count | 31 | 798 | 829 |
|  |  | % within Mother's education | 3,7% | 96,3% | 100,0% |
| Total | | Count | 111 | 3692 | 3803 |
|  |  | % within Mother's education | 2,9% | 97,1% | 100,0% |

| **Chi-Square Tests** | | | | | |
| --- | --- | --- | --- | --- | --- |
|  | Value | df | Asymptotic Significance (2-sided) | Exact Sig. (2-sided) | Exact Sig. (1-sided) |
| Pearson Chi-Square | 2,520^a^ | 1 | ,112 |  |  |
| Continuity Correction^b^ | 2,163 | 1 | ,141 |  |  |
| Likelihood Ratio | 2,377 | 1 | ,123 |  |  |
| Fisher's Exact Test |  |  |  | ,128 | ,073 |
| Linear-by-Linear Association | 2,519 | 1 | ,112 |  |  |
| N of Valid Cases | 3803 |  |  |  |  |
| a. 0 cells (,0%) have expected count less than 5. The minimum expected count is 24,20. | | | | | |
| b. Computed only for a 2x2 table | | | | | |

| **Risk Estimate** | | | |
| --- | --- | --- | --- |
|  | Value | 95% Confidence Interval | |
|  |  | Lower | Upper |
| Odds Ratio for Mother's education (High school or above / Middle school or below) | ,712 | ,467 | 1,085 |
| For cohort E-cigarette use = Yes | ,719 | ,479 | 1,081 |
| For cohort E-cigarette use = No | 1,011 | ,996 | 1,026 |
| N of Valid Cases | 3803 |  |  |

**Family e-cig use * E-cigarette use**

| **Crosstab** | | | | | |
| --- | --- | --- | --- | --- | --- |
|  | | | E-cigarette use | | Total |
|  |  |  | Yes | No |  |
| Family e-cig use | Yes | Count | 50 | 442 | 492 |
|  |  | % within Family e-cig use | 10,2% | 89,8% | 100,0% |
|  | No | Count | 61 | 3479 | 3540 |
|  |  | % within Family e-cig use | 1,7% | 98,3% | 100,0% |
| Total | | Count | 111 | 3921 | 4032 |
|  |  | % within Family e-cig use | 2,8% | 97,2% | 100,0% |

| **Chi-Square Tests** | | | | | |
| --- | --- | --- | --- | --- | --- |
|  | Value | df | Asymptotic Significance (2-sided) | Exact Sig. (2-sided) | Exact Sig. (1-sided) |
| Pearson Chi-Square | 114,920^a^ | 1 | ,000 |  |  |
| Continuity Correction^b^ | 111,789 | 1 | ,000 |  |  |
| Likelihood Ratio | 76,679 | 1 | ,000 |  |  |
| Fisher's Exact Test |  |  |  | ,000 | ,000 |
| Linear-by-Linear Association | 114,892 | 1 | ,000 |  |  |
| N of Valid Cases | 4032 |  |  |  |  |
| a. 0 cells (,0%) have expected count less than 5. The minimum expected count is 13,54. | | | | | |
| b. Computed only for a 2x2 table | | | | | |

| **Risk Estimate** | | | |
| --- | --- | --- | --- |
|  | Value | 95% Confidence Interval | |
|  |  | Lower | Upper |
| Odds Ratio for Family e-cig use (Yes / No) | 6,452 | 4,382 | 9,498 |
| For cohort E-cigarette use = Yes | 5,898 | 4,107 | 8,469 |
| For cohort E-cigarette use = No | ,914 | ,887 | ,942 |
| N of Valid Cases | 4032 |  |  |

**Family smoking * E-cigarette use**

| **Crosstab** | | | | | |
| --- | --- | --- | --- | --- | --- |
|  | | | E-cigarette use | | Total |
|  |  |  | Yes | No |  |
| Family smoking | Someone | Count | 112 | 3929 | 4041 |
|  |  | % within Family smoking | 2,8% | 97,2% | 100,0% |
|  | No one | Count | 2 | 12 | 14 |
|  |  | % within Family smoking | 14,3% | 85,7% | 100,0% |
| Total | | Count | 114 | 3941 | 4055 |
|  |  | % within Family smoking | 2,8% | 97,2% | 100,0% |

| **Chi-Square Tests** | | | | | |
| --- | --- | --- | --- | --- | --- |
|  | Value | df | Asymptotic Significance (2-sided) | Exact Sig. (2-sided) | Exact Sig. (1-sided) |
| Pearson Chi-Square | 6,770^a^ | 1 | ,009 |  |  |
| Continuity Correction^b^ | 3,211 | 1 | ,073 |  |  |
| Likelihood Ratio | 3,511 | 1 | ,061 |  |  |
| Fisher's Exact Test |  |  |  | ,057 | ,057 |
| Linear-by-Linear Association | 6,768 | 1 | ,009 |  |  |
| N of Valid Cases | 4055 |  |  |  |  |
| a. 1 cells (25,0%) have expected count less than 5. The minimum expected count is ,39. | | | | | |
| b. Computed only for a 2x2 table | | | | | |

| **Risk Estimate** | | | |
| --- | --- | --- | --- |
|  | Value | 95% Confidence Interval | |
|  |  | Lower | Upper |
| Odds Ratio for Family smoking (Someone / No one) | ,171 | ,038 | ,773 |
| For cohort E-cigarette use = Yes | ,194 | ,053 | ,709 |
| For cohort E-cigarette use = No | 1,134 | ,916 | 1,405 |
| N of Valid Cases | 4055 |  |  |

| **Case Processing Summary** | | | | | | |
| --- | --- | --- | --- | --- | --- | --- |
|  | Cases | | | | | |
|  | Valid | | Missing | | Total | |
|  | N | Percent | N | Percent | N | Percent |
| Any combustible product (Current) * E-cigarette use | 3876 | 94,6% | 220,000 | 5,4% | 4096 | 100,0% |

| **Any combustible product (Current) * E-cigarette use Crosstabulation** | | | | | |
| --- | --- | --- | --- | --- | --- |
|  | | | E-cigarette use | | Total |
|  |  |  | Yes | No |  |
| Any combustible product (Current) | Yes | Count | 64 | 448 | 512 |
|  |  | % within Any combustible product (Current) | 12,5% | 87,5% | 100,0% |
|  | No | Count | 41 | 3323 | 3364 |
|  |  | % within Any combustible product (Current) | 1,2% | 98,8% | 100,0% |
| Total | | Count | 105 | 3771 | 3876 |
|  |  | % within Any combustible product (Current) | 2,7% | 97,3% | 100,0% |

| **Chi-Square Tests** | | | | | |
| --- | --- | --- | --- | --- | --- |
|  | Value | df | Asymptotic Significance (2-sided) | Exact Sig. (2-sided) | Exact Sig. (1-sided) |
| Pearson Chi-Square | 214,573^a^ | 1 | ,000 |  |  |
| Continuity Correction^b^ | 210,314 | 1 | ,000 |  |  |
| Likelihood Ratio | 136,225 | 1 | ,000 |  |  |
| Fisher's Exact Test |  |  |  | ,000 | ,000 |
| Linear-by-Linear Association | 214,518 | 1 | ,000 |  |  |
| N of Valid Cases | 3876 |  |  |  |  |
| a. 0 cells (,0%) have expected count less than 5. The minimum expected count is 13,87. | | | | | |
| b. Computed only for a 2x2 table | | | | | |

| **Risk Estimate** | | | |
| --- | --- | --- | --- |
|  | Value | 95% Confidence Interval | |
|  |  | Lower | Upper |
| Odds Ratio for Any combustible product (Current) (Yes / No) | 11,578 | 7,728 | 17,347 |
| For cohort E-cigarette use = Yes | 10,256 | 7,008 | 15,011 |
| For cohort E-cigarette use = No | ,886 | ,857 | ,915 |
| N of Valid Cases | 3876 |  |  |

**Logistic Regression**

| **Case Processing Summary** | | | |
| --- | --- | --- | --- |
| Unweighted Cases^a^ | | N | Percent |
| Selected Cases | Included in Analysis | 3523 | 86,0 |
|  | Missing Cases | 573 | 14,0 |
|  | Total | 4096 | 100,0 |
| Unselected Cases | | 0 | ,0 |
| Total | | 4096 | 100,0 |
| a. If weight is in effect, see classification table for the total number of cases. | | | |

| **Dependent Variable Encoding** | |
| --- | --- |
| Original Value | Internal Value |
| Yes | 0 |
| No | 1 |

| **Categorical Variables Codings** | | | | |
| --- | --- | --- | --- | --- |
|  | | Frequency | Parameter coding | |
|  |  |  | (1) | (2) |
| Age | 13 years old | 1272 | ,000 | ,000 |
|  | 14 years old | 1229 | 1,000 | ,000 |
|  | 15 years old | 1022 | ,000 | 1,000 |
| Gender | Male | 1773 | ,000 |  |
|  | Female | 1750 | 1,000 |  |
| Family e-cig use | Yes | 446 | ,000 |  |
|  | No | 3077 | 1,000 |  |
| Any combustible product (Current) | Yes | 470 | ,000 |  |
|  | No | 3053 | 1,000 |  |
| Father's education | High school or above | 2601 | ,000 |  |
|  | Middle school or below | 922 | 1,000 |  |

**Block 0: Beginning Block**

| **Classification Table^a,b^** | | | | | |
| --- | --- | --- | --- | --- | --- |
|  | Observed | | Predicted | | |
|  |  |  | E-cigarette use | | Percentage Correct |
|  |  |  | Yes | No |  |
| Step 0 | E-cigarette use | Yes | 0 | 95 | ,0 |
|  |  | No | 0 | 3437 | 100,0 |
|  | Overall Percentage | |  |  | 97,3 |
| a. Constant is included in the model. | | | | | |
| b. The cut value is ,500 | | | | | |

| **Variables in the Equation** | | | | | | | |
| --- | --- | --- | --- | --- | --- | --- | --- |
|  | | B | S.E. | Wald | df | Sig. | Exp(B) |
| Step 0 | Constant | 3,588 | ,104 | 1190,846 | 1 | ,000 | 36,159 |

| **Variables not in the Equation** | | | | | |
| --- | --- | --- | --- | --- | --- |
|  | | | Score | df | Sig. |
| Step 0 | Variables | Age | 29,051 | 2 | ,000 |
|  |  | Age(1) | ,160 | 1 | ,689 |
|  |  | Age(2) | 20,925 | 1 | ,000 |
|  |  | Gender(1) | 23,184 | 1 | ,000 |
|  |  | Father's education(1) | 2,435 | 1 | ,119 |
|  |  | Any combustible product (Current)(1) | 190,153 | 1 | ,000 |
|  |  | Family e-cig use(1) | 96,512 | 1 | ,000 |
|  | Overall Statistics | | 295,546 | 6 | ,000 |

**Block 1: Method = Enter**

| **Omnibus Tests of Model Coefficients** | | | | |
| --- | --- | --- | --- | --- |
|  | | Chi-square | df | Sig. |
| Step 1 | Step | 200,663 | 6 | ,000 |
|  | Block | 200,663 | 6 | ,000 |
|  | Model | 200,663 | 6 | ,000 |

| **Model Summary** | | | |
| --- | --- | --- | --- |
| Step | -2 Log likelihood | Cox & Snell R Square | Nagelkerke R Square |
| 1 | 674,236^a^ | ,055 | ,252 |
| a. Estimation terminated at iteration number 8 because parameter estimates changed by less than ,001. | | | |

| **Hosmer and Lemeshow Test** | | | |
| --- | --- | --- | --- |
| Step | Chi-square | df | Sig. |
| 1 | 6,826 | 8 | ,556 |

| **Contingency Table for Hosmer and Lemeshow Test** | | | | | | |
| --- | --- | --- | --- | --- | --- | --- |
|  | | E-cigarette use = Yes | | E-cigarette use = No | | Total |
|  |  | Observed | Expected | Observed | Expected |  |
| Step 1 | 1 | 59 | 56,243 | 307 | 309,536 | 366 |
|  | 2 | 16 | 14,465 | 328 | 329,541 | 344 |
|  | 3 | 8 | 6,665 | 347 | 348,335 | 355 |
|  | 4 | 3 | 3,384 | 219 | 218,921 | 222 |
|  | 5 | 2 | 4,113 | 330 | 328,118 | 332 |
|  | 6 | 4 | 1,957 | 234 | 235,871 | 238 |
|  | 7 | 0 | 1,864 | 269 | 266,780 | 269 |
|  | 8 | 2 | 2,606 | 417 | 416,956 | 420 |
|  | 9 | 1 | 2,413 | 460 | 458,937 | 461 |
|  | 10 | 0 | 1,354 | 526 | 524,422 | 526 |

| **Classification Table^a^** | | | | | |
| --- | --- | --- | --- | --- | --- |
|  | Observed | | Predicted | | |
|  |  |  | E-cigarette use | | Percentage Correct |
|  |  |  | Yes | No |  |
| Step 1 | E-cigarette use | Yes | 2 | 93 | 2,5 |
|  |  | No | 4 | 3433 | 99,9 |
|  | Overall Percentage | |  |  | 97,3 |
| a. The cut value is ,500 | | | | | |

| **Variables in the Equation** | | | | | | | | | |
| --- | --- | --- | --- | --- | --- | --- | --- | --- | --- |
|  | | B | S.E. | Wald | df | Sig. | Exp(B) | 95% C.I.for EXP(B) | |
|  |  |  |  |  |  |  |  | Lower | Upper |
| Step 1^a^ | Age |  |  | 9,862 | 2 | ,007 |  |  |  |
|  | Age(1) | -,696 | ,341 | 4,158 | 1 | ,041 | ,499 | ,255 | ,973 |
|  | Age(2) | -1,053 | ,338 | 9,709 | 1 | ,002 | ,349 | ,180 | ,677 |
|  | Gender(1) | ,942 | ,252 | 13,946 | 1 | ,000 | 2,564 | 1,564 | 4,203 |
|  | Father's education(1) | -,233 | ,236 | ,976 | 1 | ,323 | ,792 | ,499 | 1,258 |
|  | Any combustible product (Current)(1) | 2,060 | ,230 | 80,527 | 1 | ,000 | 7,849 | 5,004 | 12,309 |
|  | Family e-cig use(1) | 1,745 | ,229 | 57,876 | 1 | ,000 | 5,724 | 3,652 | 8,973 |
|  | Constant | 1,270 | ,363 | 12,220 | 1 | ,000 | 3,561 |  |  |
| a. Variable(s) entered on step 1: Age, Gender, Father's education, Any combustible product (Current), Family e-cig use. | | | | | | | | | |

NB.: For the Age variable, SPSS produced adjusted odds ratios for the inverse association (1/x), so when this was presented in the Results, the multiplicative inverse numbers are shown.

**Crosstabs**

| **Case Processing Summary** | | | | | | |
| --- | --- | --- | --- | --- | --- | --- |
|  | Cases | | | | | |
|  | Valid | | Missing | | Total | |
|  | N | Percent | N | Percent | N | Percent |
| Age * Susceptibility to smoking? | 2510 | 99,3% | 17,426 | 0,7% | 2527,426 | 100,0% |
| Gender * Susceptibility to smoking? | 2510 | 99,3% | 17,426 | 0,7% | 2527,426 | 100,0% |
| Pocket money * Susceptibility to smoking? | 2504^a^ | 99,1% | 23,426 | 0,9% | 2527,426 | 100,0% |
| Father's education * Susceptibility to smoking? | 2297^a^ | 90,9% | 230,426 | 9,1% | 2527,426 | 100,0% |
| Mother's education * Susceptibility to smoking? | 2350^a^ | 93,0% | 177,426 | 7,0% | 2527,426 | 100,0% |
| Ever e-cig use * Susceptibility to smoking? | 2508^a^ | 99,2% | 19,426 | 0,8% | 2527,426 | 100,0% |
| Family e-cig use * Susceptibility to smoking? | 2502^a^ | 99,0% | 25,426 | 1,0% | 2527,426 | 100,0% |
| Family smoking * Susceptibility to smoking? | 2510^a^ | 99,3% | 17,426 | 0,7% | 2527,426 | 100,0% |
| Ever shisha use * Susceptibility to smoking? | 2481^a^ | 98,2% | 46,426 | 1,8% | 2527,426 | 100,0% |
| a. Number of valid cases is different from the total count in the crosstabulation table because the cell counts have been rounded. | | | | | | |

**Age * Susceptibility to smoking?**

| **Crosstab** | | | | | |
| --- | --- | --- | --- | --- | --- |
|  | | | Susceptibility to smoking? | | Total |
|  |  |  | 1 | 2 |  |
| Age | 13 years old | Count | 105 | 993 | 1098 |
|  |  | % within Age | 9,6% | 90,4% | 100,0% |
|  | 14 years old | Count | 133 | 732 | 865 |
|  |  | % within Age | 15,4% | 84,6% | 100,0% |
|  | 15 years old | Count | 85 | 462 | 547 |
|  |  | % within Age | 15,5% | 84,5% | 100,0% |
| Total | | Count | 323 | 2187 | 2510 |
|  |  | % within Age | 12,9% | 87,1% | 100,0% |

| **Chi-Square Tests** | | | |
| --- | --- | --- | --- |
|  | Value | df | Asymptotic Significance (2-sided) |
| Pearson Chi-Square | 19,030^a^ | 2 | ,000 |
| Likelihood Ratio | 19,503 | 2 | ,000 |
| Linear-by-Linear Association | 15,159 | 1 | ,000 |
| N of Valid Cases | 2510 |  |  |
| a. 0 cells (,0%) have expected count less than 5. The minimum expected count is 70,39. | | | |

| **Risk Estimate** | |
| --- | --- |
|  | Value |
| Odds Ratio for Age (13 years old / 14 years old) | ^a^ |
| a. Risk Estimate statistics cannot be computed. They are only computed for a 2*2 table without empty cells. | |

**Gender * Susceptibility to smoking?**

| **Crosstab** | | | | | |
| --- | --- | --- | --- | --- | --- |
|  | | | Susceptibility to smoking? | | Total |
|  |  |  | 1 | 2 |  |
| Gender | Male | Count | 139 | 1096 | 1235 |
|  |  | % within Gender | 11,3% | 88,7% | 100,0% |
|  | Female | Count | 184 | 1091 | 1275 |
|  |  | % within Gender | 14,4% | 85,6% | 100,0% |
| Total | | Count | 323 | 2187 | 2510 |
|  |  | % within Gender | 12,9% | 87,1% | 100,0% |

| **Chi-Square Tests** | | | | | |
| --- | --- | --- | --- | --- | --- |
|  | Value | df | Asymptotic Significance (2-sided) | Exact Sig. (2-sided) | Exact Sig. (1-sided) |
| Pearson Chi-Square | 5,645^a^ | 1 | ,018 |  |  |
| Continuity Correction^b^ | 5,365 | 1 | ,021 |  |  |
| Likelihood Ratio | 5,664 | 1 | ,017 |  |  |
| Fisher's Exact Test |  |  |  | ,020 | ,010 |
| Linear-by-Linear Association | 5,643 | 1 | ,018 |  |  |
| N of Valid Cases | 2510 |  |  |  |  |
| a. 0 cells (,0%) have expected count less than 5. The minimum expected count is 158,93. | | | | | |
| b. Computed only for a 2x2 table | | | | | |

| **Risk Estimate** | | | |
| --- | --- | --- | --- |
|  | Value | 95% Confidence Interval | |
|  |  | Lower | Upper |
| Odds Ratio for Gender (Male / Female) | ,752 | ,594 | ,952 |
| For cohort Susceptibility to smoking? = 1 | ,780 | ,635 | ,958 |
| For cohort Susceptibility to smoking? = 2 | 1,037 | 1,006 | 1,069 |
| N of Valid Cases | 2510 |  |  |

**Pocket money * Susceptibility to smoking?**

| **Crosstab** | | | | | |
| --- | --- | --- | --- | --- | --- |
|  | | | Susceptibility to smoking? | | Total |
|  |  |  | 1 | 2 |  |
| Pocket money | No money | Count | 12 | 143 | 155 |
|  |  | % within Pocket money | 7,7% | 92,3% | 100,0% |
|  | 1-9€ | Count | 158 | 1329 | 1487 |
|  |  | % within Pocket money | 10,6% | 89,4% | 100,0% |
|  | 10-19€ | Count | 121 | 551 | 672 |
|  |  | % within Pocket money | 18,0% | 82,0% | 100,0% |
|  | >20€ | Count | 32 | 158 | 190 |
|  |  | % within Pocket money | 16,8% | 83,2% | 100,0% |
| Total | | Count | 323 | 2181 | 2504 |
|  |  | % within Pocket money | 12,9% | 87,1% | 100,0% |

| **Chi-Square Tests** | | | |
| --- | --- | --- | --- |
|  | Value | df | Asymptotic Significance (2-sided) |
| Pearson Chi-Square | 28,739^a^ | 3 | ,000 |
| Likelihood Ratio | 27,984 | 3 | ,000 |
| Linear-by-Linear Association | 23,074 | 1 | ,000 |
| N of Valid Cases | 2504 |  |  |
| a. 0 cells (,0%) have expected count less than 5. The minimum expected count is 19,99. | | | |

| **Risk Estimate** | |
| --- | --- |
|  | Value |
| Odds Ratio for Pocket money (No money / 1-9€) | ^a^ |
| a. Risk Estimate statistics cannot be computed. They are only computed for a 2*2 table without empty cells. | |

**Father's education * Susceptibility to smoking?**

| **Crosstab** | | | | | |
| --- | --- | --- | --- | --- | --- |
|  | | | Susceptibility to smoking? | | Total |
|  |  |  | 1 | 2 |  |
| Father's education | High school or above | Count | 230 | 1515 | 1745 |
|  |  | % within Father's education | 13,2% | 86,8% | 100,0% |
|  | Middle school or below | Count | 65 | 487 | 552 |
|  |  | % within Father's education | 11,8% | 88,2% | 100,0% |
| Total | | Count | 295 | 2002 | 2297 |
|  |  | % within Father's education | 12,8% | 87,2% | 100,0% |

| **Chi-Square Tests** | | | | | |
| --- | --- | --- | --- | --- | --- |
|  | Value | df | Asymptotic Significance (2-sided) | Exact Sig. (2-sided) | Exact Sig. (1-sided) |
| Pearson Chi-Square | ,740^a^ | 1 | ,390 |  |  |
| Continuity Correction^b^ | ,619 | 1 | ,431 |  |  |
| Likelihood Ratio | ,752 | 1 | ,386 |  |  |
| Fisher's Exact Test |  |  |  | ,422 | ,217 |
| Linear-by-Linear Association | ,739 | 1 | ,390 |  |  |
| N of Valid Cases | 2297 |  |  |  |  |
| a. 0 cells (,0%) have expected count less than 5. The minimum expected count is 70,89. | | | | | |
| b. Computed only for a 2x2 table | | | | | |

| **Risk Estimate** | | | |
| --- | --- | --- | --- |
|  | Value | 95% Confidence Interval | |
|  |  | Lower | Upper |
| Odds Ratio for Father's education (High school or above / Middle school or below) | 1,137 | ,848 | 1,526 |
| For cohort Susceptibility to smoking? = 1 | 1,119 | ,865 | 1,449 |
| For cohort Susceptibility to smoking? = 2 | ,984 | ,950 | 1,020 |
| N of Valid Cases | 2297 |  |  |

**Mother's education * Susceptibility to smoking?**

| **Crosstab** | | | | | |
| --- | --- | --- | --- | --- | --- |
|  | | | Susceptibility to smoking? | | Total |
|  |  |  | 1 | 2 |  |
| Mother's education | High school or above | Count | 257 | 1642 | 1899 |
|  |  | % within Mother's education | 13,5% | 86,5% | 100,0% |
|  | Middle school or below | Count | 52 | 399 | 451 |
|  |  | % within Mother's education | 11,5% | 88,5% | 100,0% |
| Total | | Count | 309 | 2041 | 2350 |
|  |  | % within Mother's education | 13,1% | 86,9% | 100,0% |

| **Chi-Square Tests** | | | | | |
| --- | --- | --- | --- | --- | --- |
|  | Value | df | Asymptotic Significance (2-sided) | Exact Sig. (2-sided) | Exact Sig. (1-sided) |
| Pearson Chi-Square | 1,281^a^ | 1 | ,258 |  |  |
| Continuity Correction^b^ | 1,112 | 1 | ,292 |  |  |
| Likelihood Ratio | 1,318 | 1 | ,251 |  |  |
| Fisher's Exact Test |  |  |  | ,278 | ,146 |
| Linear-by-Linear Association | 1,280 | 1 | ,258 |  |  |
| N of Valid Cases | 2350 |  |  |  |  |
| a. 0 cells (,0%) have expected count less than 5. The minimum expected count is 59,30. | | | | | |
| b. Computed only for a 2x2 table | | | | | |

| **Risk Estimate** | | | |
| --- | --- | --- | --- |
|  | Value | 95% Confidence Interval | |
|  |  | Lower | Upper |
| Odds Ratio for Mother's education (High school or above / Middle school or below) | 1,201 | ,874 | 1,650 |
| For cohort Susceptibility to smoking? = 1 | 1,174 | ,887 | 1,553 |
| For cohort Susceptibility to smoking? = 2 | ,977 | ,941 | 1,015 |
| N of Valid Cases | 2350 |  |  |

**Ever e-cig use * Susceptibility to smoking?**

| **Crosstab** | | | | | |
| --- | --- | --- | --- | --- | --- |
|  | | | Susceptibility to smoking? | | Total |
|  |  |  | 1 | 2 |  |
| Ever e-cig use | Yes | Count | 19 | 42 | 61 |
|  |  | % within Ever e-cig use | 31,1% | 68,9% | 100,0% |
|  | No | Count | 304 | 2143 | 2447 |
|  |  | % within Ever e-cig use | 12,4% | 87,6% | 100,0% |
| Total | | Count | 323 | 2185 | 2508 |
|  |  | % within Ever e-cig use | 12,9% | 87,1% | 100,0% |

| **Chi-Square Tests** | | | | | |
| --- | --- | --- | --- | --- | --- |
|  | Value | df | Asymptotic Significance (2-sided) | Exact Sig. (2-sided) | Exact Sig. (1-sided) |
| Pearson Chi-Square | 18,597^a^ | 1 | ,000 |  |  |
| Continuity Correction^b^ | 16,966 | 1 | ,000 |  |  |
| Likelihood Ratio | 14,249 | 1 | ,000 |  |  |
| Fisher's Exact Test |  |  |  | ,000 | ,000 |
| Linear-by-Linear Association | 18,590 | 1 | ,000 |  |  |
| N of Valid Cases | 2508 |  |  |  |  |
| a. 0 cells (,0%) have expected count less than 5. The minimum expected count is 7,86. | | | | | |
| b. Computed only for a 2x2 table | | | | | |

| **Risk Estimate** | | | |
| --- | --- | --- | --- |
|  | Value | 95% Confidence Interval | |
|  |  | Lower | Upper |
| Odds Ratio for Ever e-cig use (Yes / No) | 3,189 | 1,831 | 5,555 |
| For cohort Susceptibility to smoking? = 1 | 2,507 | 1,701 | 3,694 |
| For cohort Susceptibility to smoking? = 2 | ,786 | ,664 | ,931 |
| N of Valid Cases | 2508 |  |  |

**Family e-cig use * Susceptibility to smoking?**

| **Crosstab** | | | | | |
| --- | --- | --- | --- | --- | --- |
|  | | | Susceptibility to smoking? | | Total |
|  |  |  | 1 | 2 |  |
| Family e-cig use | Yes | Count | 36 | 209 | 245 |
|  |  | % within Family e-cig use | 14,7% | 85,3% | 100,0% |
|  | No | Count | 286 | 1971 | 2257 |
|  |  | % within Family e-cig use | 12,7% | 87,3% | 100,0% |
| Total | | Count | 322 | 2180 | 2502 |
|  |  | % within Family e-cig use | 12,9% | 87,1% | 100,0% |

| **Chi-Square Tests** | | | | | |
| --- | --- | --- | --- | --- | --- |
|  | Value | df | Asymptotic Significance (2-sided) | Exact Sig. (2-sided) | Exact Sig. (1-sided) |
| Pearson Chi-Square | ,806^a^ | 1 | ,369 |  |  |
| Continuity Correction^b^ | ,636 | 1 | ,425 |  |  |
| Likelihood Ratio | ,779 | 1 | ,377 |  |  |
| Fisher's Exact Test |  |  |  | ,366 | ,210 |
| Linear-by-Linear Association | ,806 | 1 | ,369 |  |  |
| N of Valid Cases | 2502 |  |  |  |  |
| a. 0 cells (,0%) have expected count less than 5. The minimum expected count is 31,53. | | | | | |
| b. Computed only for a 2x2 table | | | | | |

| **Risk Estimate** | | | |
| --- | --- | --- | --- |
|  | Value | 95% Confidence Interval | |
|  |  | Lower | Upper |
| Odds Ratio for Family e-cig use (Yes / No) | 1,187 | ,816 | 1,727 |
| For cohort Susceptibility to smoking? = 1 | 1,160 | ,842 | 1,598 |
| For cohort Susceptibility to smoking? = 2 | ,977 | ,925 | 1,031 |
| N of Valid Cases | 2502 |  |  |

**Family smoking * Susceptibility to smoking?**

| **Crosstab** | | | | | |
| --- | --- | --- | --- | --- | --- |
|  | | | Susceptibility to smoking? | | Total |
|  |  |  | 1 | 2 |  |
| Family smoking | Someone | Count | 322 | 2183 | 2505 |
|  |  | % within Family smoking | 12,9% | 87,1% | 100,0% |
|  | No one | Count | 1 | 4 | 5 |
|  |  | % within Family smoking | 20,0% | 80,0% | 100,0% |
| Total | | Count | 323 | 2187 | 2510 |
|  |  | % within Family smoking | 12,9% | 87,1% | 100,0% |

| **Chi-Square Tests** | | | | | |
| --- | --- | --- | --- | --- | --- |
|  | Value | df | Asymptotic Significance (2-sided) | Exact Sig. (2-sided) | Exact Sig. (1-sided) |
| Pearson Chi-Square | ,227^a^ | 1 | ,634 |  |  |
| Continuity Correction^b^ | ,000 | 1 | 1,000 |  |  |
| Likelihood Ratio | ,199 | 1 | ,655 |  |  |
| Fisher's Exact Test |  |  |  | ,498 | ,498 |
| Linear-by-Linear Association | ,227 | 1 | ,634 |  |  |
| N of Valid Cases | 2510 |  |  |  |  |
| a. 2 cells (50,0%) have expected count less than 5. The minimum expected count is ,64. | | | | | |
| b. Computed only for a 2x2 table | | | | | |

| **Risk Estimate** | | | |
| --- | --- | --- | --- |
|  | Value | 95% Confidence Interval | |
|  |  | Lower | Upper |
| Odds Ratio for Family smoking (Someone / No one) | ,590 | ,066 | 5,295 |
| For cohort Susceptibility to smoking? = 1 | ,643 | ,111 | 3,721 |
| For cohort Susceptibility to smoking? = 2 | 1,089 | ,703 | 1,689 |
| N of Valid Cases | 2510 |  |  |

**Logistic Regression**

| **Case Processing Summary** | | | |
| --- | --- | --- | --- |
| Unweighted Cases^a^ | | N | Percent |
| Selected Cases | Included in Analysis | 2491 | 98,6 |
|  | Missing Cases | 35 | 1,4 |
|  | Total | 2526 | 100,0 |
| Unselected Cases | | 0 | ,0 |
| Total | | 2526 | 100,0 |
| a. If weight is in effect, see classification table for the total number of cases. | | | |

| **Dependent Variable Encoding** | |
| --- | --- |
| Original Value | Internal Value |
| 1 | 0 |
| 2 | 1 |

| **Categorical Variables Codings** | | | | | |
| --- | --- | --- | --- | --- | --- |
|  | | Frequency | Parameter coding | | |
|  |  |  | (1) | (2) | (3) |
| Pocket money | No money | 167 | ,000 | ,000 | ,000 |
|  | 1-9€ | 1487 | 1,000 | ,000 | ,000 |
|  | 10-19€ | 656 | ,000 | 1,000 | ,000 |
|  | >20€ | 181 | ,000 | ,000 | 1,000 |
| Age | 13 years old | 1091 | ,000 | ,000 |  |
|  | 14 years old | 844 | 1,000 | ,000 |  |
|  | 15 years old | 556 | ,000 | 1,000 |  |
| Ever e-cig use | Yes | 60 | ,000 |  |  |
|  | No | 2431 | 1,000 |  |  |
| Gender | Male | 1204 | ,000 |  |  |
|  | Female | 1287 | 1,000 |  |  |

**Block 0: Beginning Block**

| **Classification Table^a,b^** | | | | | |
| --- | --- | --- | --- | --- | --- |
|  | Observed | | Predicted | | |
|  |  |  | Susceptibility to smoking? | | Percentage Correct |
|  |  |  | 1 | 2 |  |
| Step 0 | Susceptibility to smoking? | 1 | 0 | 321 | ,0 |
|  |  | 2 | 0 | 2178 | 100,0 |
|  | Overall Percentage | |  |  | 87,2 |
| a. Constant is included in the model. | | | | | |
| b. The cut value is ,500 | | | | | |

| **Variables in the Equation** | | | | | | | |
| --- | --- | --- | --- | --- | --- | --- | --- |
|  | | B | S.E. | Wald | df | Sig. | Exp(B) |
| Step 0 | Constant | 1,915 | ,060 | 1025,831 | 1 | ,000 | 6,784 |

| **Variables not in the Equation** | | | | | |
| --- | --- | --- | --- | --- | --- |
|  | | | Score | df | Sig. |
| Step 0 | Variables | Age | 19,155 | 2 | ,000 |
|  |  | Age(1) | 7,371 | 1 | ,007 |
|  |  | Age(2) | 4,546 | 1 | ,033 |
|  |  | Gender(1) | 5,571 | 1 | ,018 |
|  |  | Pocket money | 29,123 | 3 | ,000 |
|  |  | Pocket money(1) | 17,047 | 1 | ,000 |
|  |  | Pocket money(2) | 21,675 | 1 | ,000 |
|  |  | Pocket money(3) | 2,839 | 1 | ,092 |
|  |  | Ever e-cig use(1) | 18,317 | 1 | ,000 |
|  | Overall Statistics | | 65,849 | 7 | ,000 |

**Block 1: Method = Enter**

| **Omnibus Tests of Model Coefficients** | | | | |
| --- | --- | --- | --- | --- |
|  | | Chi-square | df | Sig. |
| Step 1 | Step | 62,050 | 7 | ,000 |
|  | Block | 62,050 | 7 | ,000 |
|  | Model | 62,050 | 7 | ,000 |

| **Model Summary** | | | |
| --- | --- | --- | --- |
| Step | -2 Log likelihood | Cox & Snell R Square | Nagelkerke R Square |
| 1 | 1854,831^a^ | ,025 | ,046 |
| a. Estimation terminated at iteration number 5 because parameter estimates changed by less than ,001. | | | |

| **Hosmer and Lemeshow Test** | | | |
| --- | --- | --- | --- |
| Step | Chi-square | df | Sig. |
| 1 | 6,968 | 7 | ,432 |

| **Contingency Table for Hosmer and Lemeshow Test** | | | | | | |
| --- | --- | --- | --- | --- | --- | --- |
|  | | Susceptibility to smoking? = 1 | | Susceptibility to smoking? = 2 | | Total |
|  |  | Observed | Expected | Observed | Expected |  |
| Step 1 | 1 | 66 | 61,869 | 187 | 191,466 | 253 |
|  | 2 | 48 | 47,019 | 216 | 217,076 | 264 |
|  | 3 | 32 | 35,650 | 203 | 200,074 | 236 |
|  | 4 | 22 | 22,769 | 140 | 139,270 | 162 |
|  | 5 | 29 | 35,400 | 226 | 218,913 | 254 |
|  | 6 | 31 | 30,317 | 248 | 248,972 | 279 |
|  | 7 | 32 | 26,174 | 218 | 223,804 | 250 |
|  | 8 | 38 | 31,348 | 306 | 311,853 | 343 |
|  | 9 | 24 | 30,547 | 434 | 426,956 | 458 |

| **Classification Table^a^** | | | | | |
| --- | --- | --- | --- | --- | --- |
|  | Observed | | Predicted | | |
|  |  |  | Susceptibility to smoking? | | Percentage Correct |
|  |  |  | 1 | 2 |  |
| Step 1 | Susceptibility to smoking? | 1 | 0 | 321 | ,0 |
|  |  | 2 | 0 | 2178 | 100,0 |
|  | Overall Percentage | |  |  | 87,2 |
| a. The cut value is ,500 | | | | | |

| **Variables in the Equation** | | | | | | | | | |
| --- | --- | --- | --- | --- | --- | --- | --- | --- | --- |
|  | | B | S.E. | Wald | df | Sig. | Exp(B) | 95% C.I.for EXP(B) | |
|  |  |  |  |  |  |  |  | Lower | Upper |
| Step 1^a^ | Age |  |  | 13,937 | 2 | ,001 |  |  |  |
|  | Age(1) | -,475 | ,142 | 11,255 | 1 | ,001 | ,622 | ,471 | ,821 |
|  | Age(2) | -,486 | ,159 | 9,365 | 1 | ,002 | ,615 | ,450 | ,840 |
|  | Gender(1) | -,324 | ,123 | 6,983 | 1 | ,008 | ,723 | ,568 | ,920 |
|  | Pocket money |  |  | 22,613 | 3 | ,000 |  |  |  |
|  | Pocket money(1) | -,321 | ,317 | 1,025 | 1 | ,311 | ,726 | ,390 | 1,350 |
|  | Pocket money(2) | -,881 | ,322 | 7,474 | 1 | ,006 | ,415 | ,221 | ,779 |
|  | Pocket money(3) | -,808 | ,364 | 4,937 | 1 | ,026 | ,446 | ,219 | ,909 |
|  | Ever e-cig use(1) | 1,117 | ,292 | 14,675 | 1 | ,000 | 3,056 | 1,726 | 5,412 |
|  | Constant | 1,825 | ,415 | 19,367 | 1 | ,000 | 6,206 |  |  |
| a. Variable(s) entered on step 1: Age, Gender, Pocket money, Ever e-cig use. | | | | | | | | | |

NB.: For the Age and Pocket money variables, SPSS produced adjusted odds ratios for the inverse association (1/x), so when these were presented in the Results, the multiplicative inverse numbers are shown.
